# Discovery, Structure–Activity Relationship and In Vitro Anticancer Activity of Small-Molecule Inhibitors of the Protein–Protein Interactions between AF9/ENL and AF4 or DOT1L

**DOI:** 10.3390/cancers15215283

**Published:** 2023-11-03

**Authors:** Xin Li, Xiaowei Wu, Shenyou Nie, Jidong Zhao, Yuan Yao, Fangrui Wu, Chandra Bhushan Mishra, Md Ashraf-Uz-Zaman, Bala Krishna Moku, Yongcheng Song

**Affiliations:** 1 Verna and Marrs McLean Department of Biochemistry and Molecular Pharmacology, Baylor College of Medicine, 1 Baylor Plaza, Houston, TX 77030, USA; xin.li2@bcm.edu (X.L.); wuxiaowei@simm.ac.cn (X.W.); nieshenyou@hotmail.com (S.N.); zhaojd1989@gmail.com (J.Z.); yuany@bcm.edu (Y.Y.); fangrui.wu01@gmail.com (F.W.); chandrabhushan.mishra@bcm.edu (C.B.M.); ashrafbd87@gmail.com (M.A.-U.-Z.); bala.moku@bcm.edu (B.K.M.); 2 Dan L. Duncan Comprehensive Cancer Center, Baylor College of Medicine, 1 Baylor Plaza, Houston, TX 77030, USA

**Keywords:** MLL-rearranged leukemia, super elongation complexes, small-molecule inhibitor, protein–protein interaction, cancer therapeutics

## Abstract

**Simple Summary:**

Chromosomal translocations involving the mixed lineage leukemia (MLL) gene generate potent fusion oncogenes and cause acute myeloid leukemia or lymphocytic leukemia, which account for ~75% infant and 5–10% child/adult acute leukemia cases with a poor prognosis (5-year survival rates < 45%). Protein–protein interactions between the most frequent MLL fusion partner proteins AF9/ENL and AF4 or histone methyltransferase DOT1L are critical to malignant gene expression and are therefore a potential drug target for cancer. Compound screening followed by medicinal chemistry studies identified several novel small-molecule inhibitors showing strong inhibition of these protein–protein interactions, significant suppression of characteristic gene expression, and robust cellular anticancer activities with negligible cytotoxicity. These compounds are useful chemical probes for biological studies of these protein–protein interactions, as well as pharmacological leads for further drug development against MLL-rearranged and other leukemias.

**Abstract:**

Chromosomal translocations involving the mixed lineage leukemia (MLL) gene cause 5–10% acute leukemias with poor clinical outcomes. Protein–protein interactions (PPI) between the most frequent MLL fusion partner proteins AF9/ENL and AF4 or histone methyltransferase DOT1L are drug targets for MLL-rearranged (MLL-r) leukemia. Several benzothiophene-carboxamide compounds were identified as novel inhibitors of these PPIs with IC_50_ values as low as 1.6 μM. Structure–activity relationship studies of 77 benzothiophene and related indole and benzofuran compounds show that a 4-piperidin-1-ylphenyl or 4-pyrrolidin-1-ylphenyl substituent is essential for the activity. The inhibitors suppressed expression of MLL target genes HoxA9, Meis1 and Myc, and selectively inhibited proliferation of MLL-r and other acute myeloid leukemia cells with EC_50_ values as low as 4.7 μM. These inhibitors are useful chemical probes for biological studies of AF9/ENL, as well as pharmacological leads for further drug development against MLL-r and other leukemias.

## 1. Introduction

Acute leukemias in ~75% infant and 5–10% child/adult patients are caused by chromosomal translocations of mixed lineage leukemia (MLL, also known as MLL1 or KMT2A) gene, which generate oncogenic fusion MLL (onco-MLL). MLL-rearranged (MLL-r) leukemias can be clinically characterized as acute lymphocytic leukemia (ALL) or acute myeloid leukemia (AML) with a poor prognosis [1,2,3,4,5]. Five-year survival rates for MLL-r ALL patients are <40%, in contrast to ~90% for other pediatric ALLs [6,7,8,9,10]. The situation is even worse for very young patients with five-year survivals of <20% [9]. MLL-r AML has 5-year survival rates of ~45%, similar to other AMLs [11,12]. More effective and/or less toxic targeted therapies are needed for patients with MLL-r leukemias [13,14,15,16].

The onco-MLL in these MLL-r leukemias consists of the N-terminal ~1400 amino acid residues of MLL, which function as a transcription factor and recognize MLL-target genes, in-frame fused with one of >70 other proteins [17,18,19]. However, only several fusion partners are predominant, including transcription cofactor AF4 (~35%), AF9 (25%) and its paralog ENL (10%) [1,2,11,17]. These proteins associate with each other and recruit other proteins (e.g., positive transcription elongation factor or P-TEFb) to form super elongation complexes (SEC) [20,21,22], which promote gene transcription elongation. Thus, certain MLL target genes, such as HoxA9 and Myc [23,24,25], are constitutively or excessively expressed, causing leukemia.

The C-terminal AHD domain (~70 residues) of AF9 and highly homologous ENL is a novel and validated drug target for MLL-r leukemia [11,26]. Although it is disordered by itself, AF9/ENL AHD forms a structured protein complex with a consensus peptide segment of LxVxIxLxxV/L in AF4 or its paralog AFF4 with a high affinity [27,28]. Such protein–protein interaction (PPI) is essential for the oncoprotein MLL-AF9/-ENL or MLL-AF4 to recruit SEC for aberrant gene expression. Moreover, similar interactions between AF9/ENL AHD and histone-H3 lysine-79 (H3K79) methyltransferase DOT1L (which also contains several LxVxIxLxxV/L sequences) can recruit DOT1L to MLL target gene loci, causing genome-wide H3K79 hypermethylation, which has been observed and characteristic to MLL-r leukemia [29]. Knockdown or pharmacological inhibition of DOT1L was found to selectively inhibit MLL-r leukemia in cells, animal models and clinical trials [30,31,32]. Therefore, disruption of the PPIs between AF9/ENL and AF4/AFF4 or DOT1L is a potentially useful therapy for MLL-r leukemia by suppressing SEC-mediated gene expression and DOT1L-caused H3K79 methylation [26].

**Chart 1:**
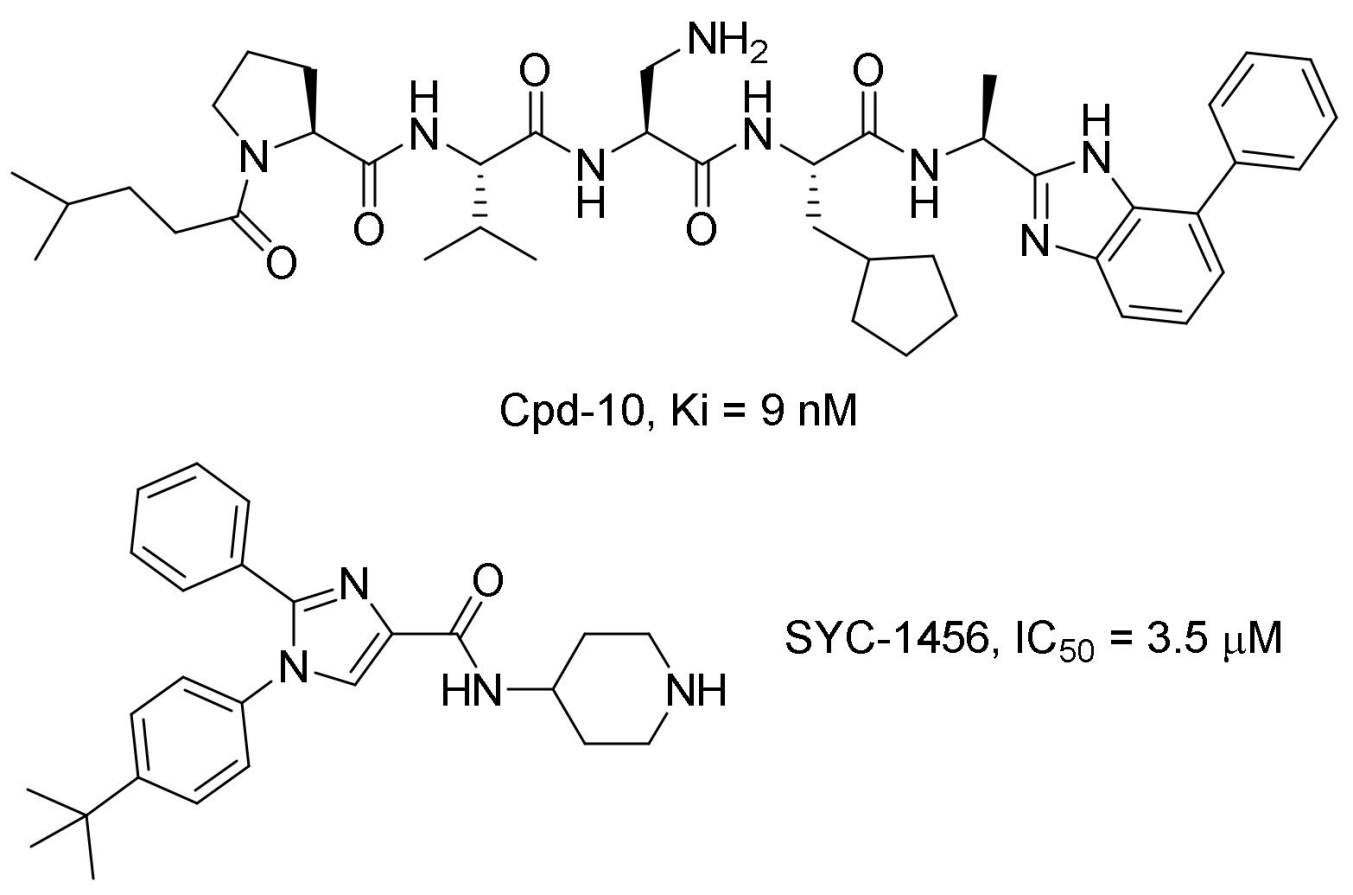
Known inhibitors of the AF9/ENL-DOT1L interaction.

Two series of compounds have been reported to inhibit the AF9/ENL-DOT1L interactions, including our previously disclosed compound SYC-1456 [26] and a series of 7-mer peptidomimetic compounds derived from DOT1L, such as Cpd-10 [33,34] (Chart 1). These compounds can inhibit AF9-DOT1L and other related PPIs and selectively suppress aberrant gene expression and cell proliferation of MLL-r leukemia, showing on-target activities, as well as a need for additional inhibitor discovery and development. Here, we report the discovery, synthesis, structure–activity relationships (SAR) and biological activities of a new chemo-type of inhibitors of the PPIs between AF9/ENL and DOT1L or AF4.

## 2. Results and Discussion

### 2.1. Discovery of Novel Inhibitors of the AF9-DOT1L Interaction

An AlphaLisa assay we previously developed [26] was used for compound screening for inhibitors of the AF9-DOT1L interaction. The PPI takes the DOT1L-peptide coated donor beads and AF9 AHD coated acceptor beads together. The donor beads are illuminated with a laser beam (680 nm) to generate singlet oxygen radicals, which activate the adjacent acceptor beads to produce luminescence at 615 nm. When a compound can disrupt such PPI and separate the donor and acceptor beads, the highly unstable radicals are rapidly quenched by water without generating luminescence. With this method, indole-carboxamide compounds **1** and **2** were found to be novel inhibitors of the AF9-DOT1L interaction, which can inhibit the PPI dose-dependently with an IC_50_ value of 3.3 and 4.5 μM, respectively (Table 1 and Supplementary Materials Figure S1), which showed comparable activities to SYC-1456 (IC_50_: 3.5 μM) [26]. Moreover, another reported inhibitor, Cpd-10 (Chart 1), was synthesized and found to exhibit an IC_50_ of 2.1 μM in our AlphaLisa assay conditions (Figure S1). These results support medicinal chemistry optimization based on the novel chemical scaffold in compounds **1** and **2**.

### 2.2. Synthesis

Medicinal chemistry based on the structures of these two compounds was next performed to investigate structure–activity relationships, as well as to find compounds with improved potency. Three series of indole- and closely related benzothiophene- and benzofuran-carboxamide compounds were synthesized for the study.

**Scheme 1:**
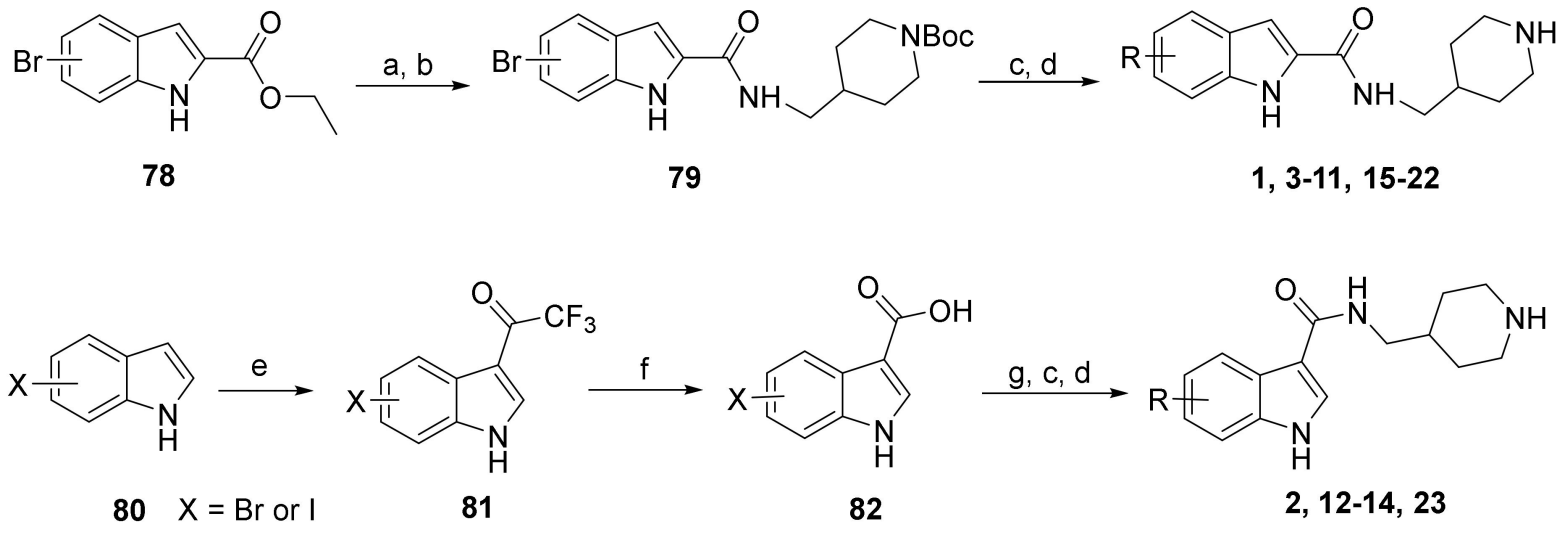
Synthesis of compounds **1**–**23**. *Reagents and conditions*: (a) NaOH, THF-H_2_O, 50 °C, 5 h; (b) 1-[bis(dimethylamino)methylene]-1*H*-1,2,3-triazolo[4,5-b]pyridinium 3-oxide hexafluorophosphate, *N*,*N*-diisopropylethylamine, DMF, 4-aminomethyl-1-Boc-piperidine, 12 h; (c) aryl boronic acid or aryl 4,4,5,5-tetramethyl-1,3,2-dioxaborolane, tetrakis(triphenylphosphine)palladium, Na_2_CO_3_, 1,4-dioxane-H_2_O, 100 °C; (d) HCl (4 N in 1,4-dioxane), CH_2_Cl_2_, 0 °C; (e) trifluoroacetic anhydride, 0 °C to room temperature, 2 h; (f) NaOH (20% aq.), 80 °C, 2 days; (g) 4-aminomethyl-1-Boc-piperidine, *N*-(3-dimethylaminopropyl)-*N*′-ethylcarbodiimide, 1-hydroxybenzotriazole, triethylamine, CH_2_Cl_2_.

The general synthesis of the indole-carboxamide compounds is shown in Scheme 1. A 5- or 6-bromo-substituted indole-2-carboxylic acid ethyl ester **78** was hydrolyzed and then reacted with *tert*-butyloxycarbonyl (BOC)-protected 4-aminomethylpiperidine to give an indole-2-carboxamide intermediate compound **79**, which was subjected to a Suzuki coupling reaction, followed by deprotection of the BOC, to give the target compounds **1**, **3**–**11** and **15**–**22**. To synthesize indole-3-carboxamide compounds, 5- or 6-bromo- or iodo-substituted indole **80** was reacted with trifluoroacetic anhydride to give 3-trifluoacetyl-indole **81**, which was hydrolyzed to provide the corresponding indole-3-carboxylic acid **82**. A similar amidation and Suzuki coupling reaction for compound **82** gave, upon BOC deprotection, the target compounds **2**, **12**–**14** and **23**.

**Scheme 2:**
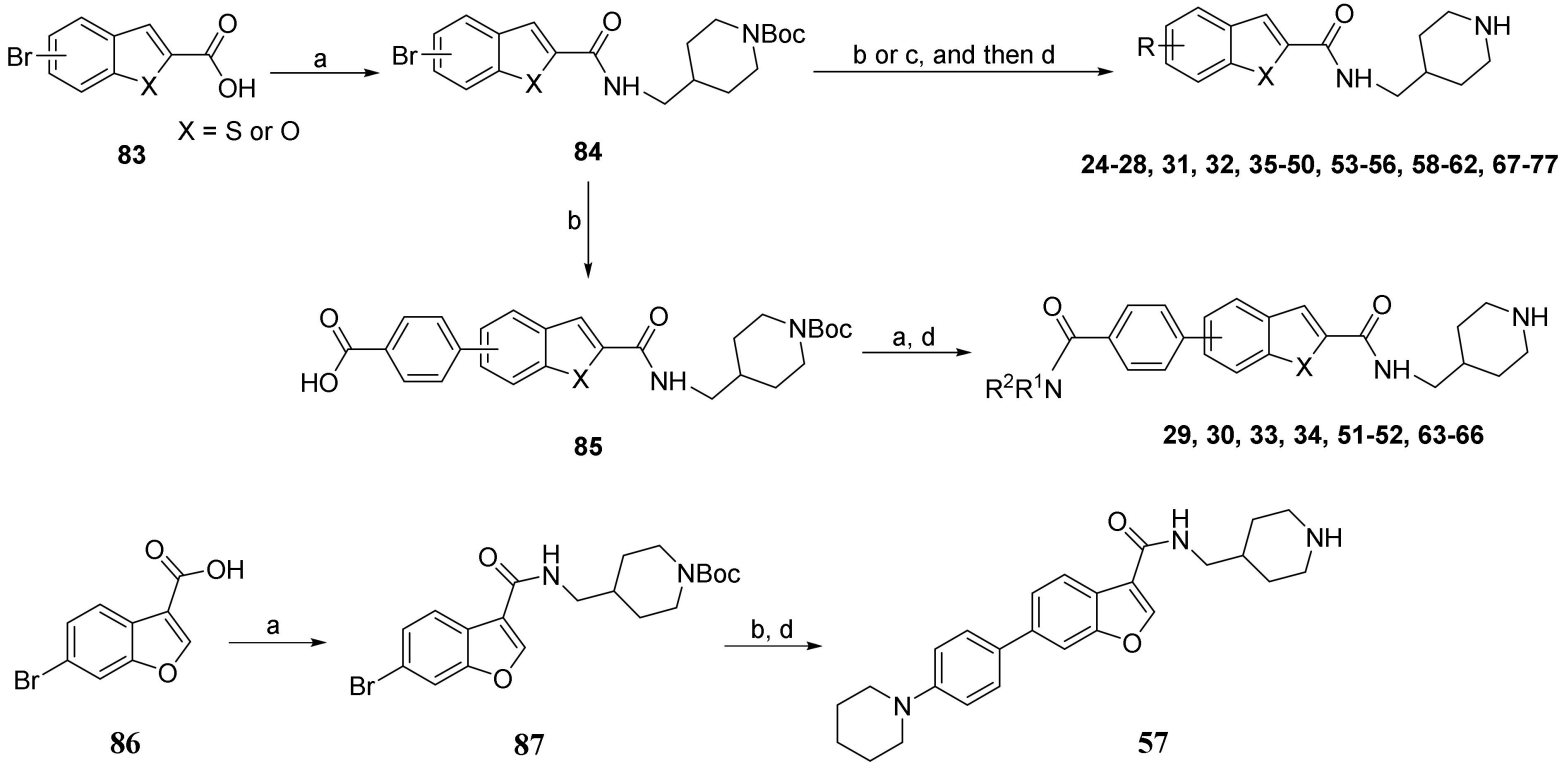
Synthesis of compounds **24**–**77**. *Reagents and conditions*: (a) 1-[bis(dimethylamino)methylene]-1*H*-1,2,3-triazolo[4,5-b]pyridinium 3-oxide hexafluorophosphate, *N*,*N*-diisopropylethylamine, DMF, 4-aminomethyl-1-Boc-piperidine or another amine,12 h; (b) aryl boronic acid or aryl 4,4,5,5-tetramethyl-1,3,2-dioxaborolane, tetrakis(triphenylphosphine)palladium, Na_2_CO_3_, 1,4-dioxane-H_2_O, 100 °C; (c) a amine or phenol, tris(dibenzylideneacetone)dipalladium, 2-dicyclohexylphosphino-2′,6′-dimethoxybiphenyl, *t*-BuOH, toluene, 100 °C; (d) HCl (4 N in 1,4-dioxane), CH_2_Cl_2_, 0 °C.

The synthesis of benzothiophene and benzofuran compounds is shown in Scheme 2. A bromo-substituted benzothiophene- or benzofuran-2-carboxylic acid **83** was coupled with BOC-protected 4-aminomethylpiperidine to provide **84**, which was subjected to a Suzuki coupling reaction with an aryl boronic acid/ester, or a Buchwald-Hartwig amination reaction with an amine/phenol to give, after deprotection of the BOC, the target compounds **24**–**28**, **31**, **32**, **35**–**50**, **53**–**56**, **58**–**62** and **67**–**77**. The carboxylic acid **85**, the Suzuki coupling product from compound **84**, reacted with an amine to yield, upon removal of the BOC, the final compounds **29**, **30**, **33**, **34**, **51**–**52** and **63**–**66**. Similar transformations from benzofuran-3-carboxylic acid **86** gave compound **57**.

### 2.3. Structure–Activity Relationships

Because of the high costs for the AlphaLisa assay, inhibitory activities of all compounds were first screened at 5 µM and the IC_50_ values for those exhibiting >50% inhibition were determined.

The structures and inhibitory activities of 6-substituted indole compounds, including compounds **1** and **2** (IC_50_ = 3.3 and 4.5 µM) with a piperidin-1-ylphenyl group, are summarized in Table 1. Ring-contracted compound **3** with a pyrrolidin-1-ylphenyl substituent was found to be equally potent, with an IC_50_ of 2.8 µM. Compound **4** without an *N* atom in the 6-substituent exhibited reduced activity (31% inhibition at 5 µM) and compound **5** having two *N* atoms is also weaker (40% inhibition). Replacing the piperidin-1-yl group with a thiophene in compound **6** (14% inhibition at 5 µM) significantly decreased activity. Changing to a 2-hydroxyethyl in compound **7** or to an acetyl group in **8** also resulted in a lowered potency. Compounds **9** and **10** with *meta*-substituted *tert*-butyl- and cyano-phenyl R^6^, respectively, are very weak (17% and 0% inhibition at 5 µM). Compound **11** with a smaller thiophene group at this position is almost inactive. In addition, three analogs of compound **2** with a 3-carboxylamide sidechain were synthesized. Compound **12** with a 4-pyrrolidin-1-ylphenyl R^6^ group was found to exhibit slightly improved activity with an IC_50_ of 2.9 µM. Compound **13** with another similar morpholin-4-yl group showed reduced activity (29% inhibition at 5 µM), while compound **14** with a polar aminomethyl group was almost inactive (11% inhibition at 5 µM).

Compounds **15**–**23** (Table 2) with a variety of groups at the 5-position of the indole core were investigated. Compounds **15** and **16** with the 4-piperidin-1-ylphenyl and 4-pyrrolidin-1-ylphenyl R^5^ group, respectively, showed comparable activities (IC_50_ = 3.6 and 2.7 µM) to compounds **1**–**3** and **12**. Other groups in compounds **17**–**22** showed less potent activities. While having a 4-piperidin-1-ylphenyl R^5^ group, compound **23** with a 3-carboxylamide sidechain showed reduced activity (43% inhibition at 5 µM).

Results of the above indole compounds show that in most cases, 4-piperidin-1-ylphenyl or similar 4-pyrrolidin-1-ylphenyl group contributes favorably to inhibition of the AF9-DOT1L interaction, regardless whether they are in the 6- or 5-position. However, despite their strong biochemical activities, these indole-containing inhibitors showed only modest activities to inhibit the proliferation of MLL-r leukemia cells (described below), which might be due to poor cell permeability or uptake of these compounds.

Next, a structurally similar, less polar benzothiophene core was used to replace indole. The structures and inhibitory activities of 6-substituted benzothiophene compounds are shown in Table 3. Compound **24** with a 4-piperidin-1-ylphenyl group was found to be a strong inhibitor of the AF9-DOT1L interaction with an IC_50_ of 1.6 µM, showing ~2× activity compared to the indole analog **1**. Compound **25** with a 4-pyrrolidin-1-ylphenyl had weaker activity (IC_50_ = 5.3 µM). Changing to a morpholine in compound **26** (40% inhibition at 5 µM) or a diethylamino group in **27** (25% inhibition at 5 µM) considerably reduced inhibitory activity. Adding a -CH_2_- (in compound **28**) or a carbonyl (in **29** and **30**) between the piperidinyl/pyrrolidine and phenyl groups lost activity (0–8% inhibition at 5 µM). Replacing the phenyl group with a non-aromatic piperidine ring yielded very weak compounds **31** and **32** (14% and 20% inhibition at 5 µM). Inserting a carbonylphenyl into these two compounds produced inactive compounds **33** and **34**. Compound **35** with an additional -NH- between the 4-piperidin-1-ylphenyl group and benzothiophene core was found to be a strong inhibitor (IC_50_ = 2.3 µM). Compound **36** having a 4-(4-chlorophenoxy)phenoxy R^6^ group is a moderate inhibitor (IC_50_~5 µM). In addition, a variety of substituted phenyl R^6^ groups in compounds **37**–**49** with different steric and electronic properties resulted in inactive to moderate inhibitors.

5-Substituted benzothiophene compounds **50**–**56** were synthesized and tested for SAR studies. As shown in Table 4, similar SAR results were observed with compound **50** having a 4-piperidin-1-ylphenyl R^5^ group showing the strongest activity (IC_50_ = 2.5 µM), while other compounds showed no to moderate activities.

Moreover, benzofuran compounds **57**–**77**, which possess subtle structural (e.g., angles between substituents), electronic or hydrophobic differences from indole or benzothiophene analogs, were synthesized for the SAR studies. As shown in Table 5, similar SARs were observed, but benzofuran inhibitors appeared to be less potent. The strongest benzofuran compounds **57** with a 4-piperidin-1-ylphenyl and **74** with a 4-piperidin-1-ylphenylamino R^6^ group had IC_50_ values of 7.2 and 4.6 µM, respectively. Replacing the 4-piperidin-1-ylphenyl group with a closely related morpholine (in compound **58**), piperazine (in **59**/**60**), cyclohexyl (in **61**), or diethylamino group (in **62**) significantly reduced the inhibitory activity. Activities of compounds **63**–**66** indicate that an amide-containing R^6^ group is disfavored. Other R^6^ groups in compounds **67**–**73** and **75**–**77** also showed no to modest activities against the AF9-DOT1L interaction.

### 2.4. Activity Confirmation by a Pull-Down Assay

**Figure 1:**
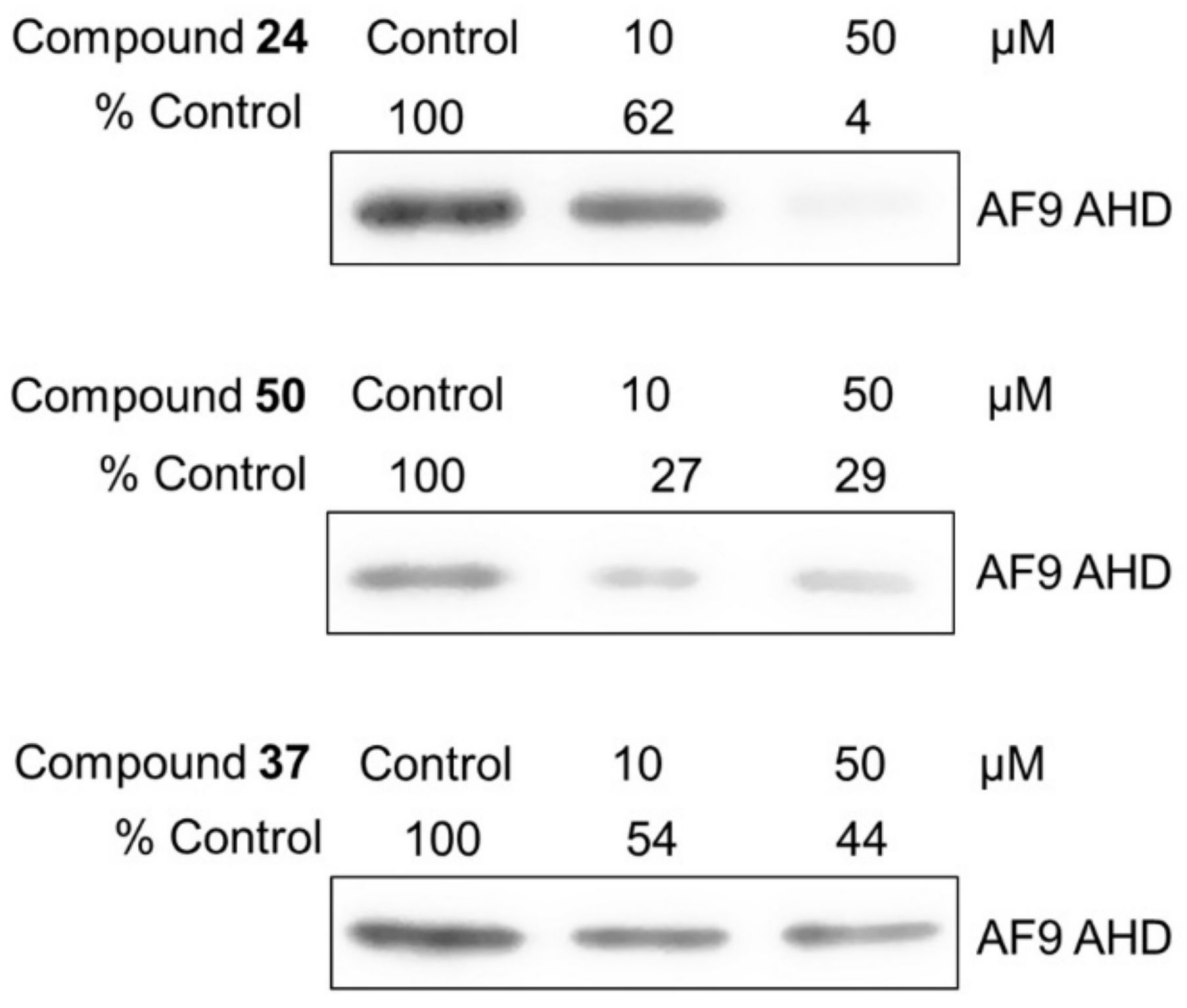
Pull-down assay indicated that compounds **24** and **50** significantly reduced the amounts of AF9 AHD bound to DOT1L peptide-coated resins in a dose-dependent manner, while inactive compound **37** did not significantly affect the AF9 AHD levels. The uncropped bolts are shown in Supplementary Materials.

A pull-down assay [26] was used to confirm that the strong inhibitors could disrupt the AF9-DOT1L interaction. Streptavidin agarose beads coated with biotinylated DOT1L peptide were used to pull-down the protein AF9 AHD in the solution, followed by thorough washing, SDS-PAGE (sodium dodecyl sulfate-polyacrylamide gel electrophoresis) and Western blotting to visualize and quantitate the protein. As shown in Figure 1, inhibitors **24** and **50** (IC_50_ = 1.6 and 2.5 µM) can dose-dependently reduce AF9 AHD, while inactive compound **37** did not significantly affect the binding of the protein to the DOT1L-coated beads even at 50 µM. This alternative, non-optical method confirmed that compounds **24** and **50** can inhibit the PPI between AF9 and DOT1L.

### 2.5. Activity to Block the AF9/ENL-AF4 Interactions

Transcription cofactor AF4, which is the most frequent (~35%) fusion protein in MLL1-r leukemia, is another binding partner of AF9/ENL [35,36,37]. The AF9/ENL-AF4 interactions are essential for the formation of SEC, which causes aberrant gene expression in MLL-r leukemia and other AMLs [20,28]. Using our previously developed AlphaLisa assays [26], selected compounds were tested for their ability to inhibit the PPIs between AF9 or ENL and AF4. As summarized in Table 6, inhibitors **16**, **24**, **25** and **50** of the AF9-DOT1L interaction (IC_50_ = 1.6–5.3 µM) were found to exhibit comparable activities to block the AF9-AF4 and ENL-AF4 interactions with IC_50_ values of 1.9–7.9 µM, while inactive compounds **22** and **37** did not inhibit these PPIs. These results are consistent with our previous finding that an inhibitor of the AF9-DOT1L interaction is broadly active against PPIs between AF9/ENL and AF4 [26].

### 2.6. Activity to Suppress Oncogenic Gene Expression

**Figure 2:**
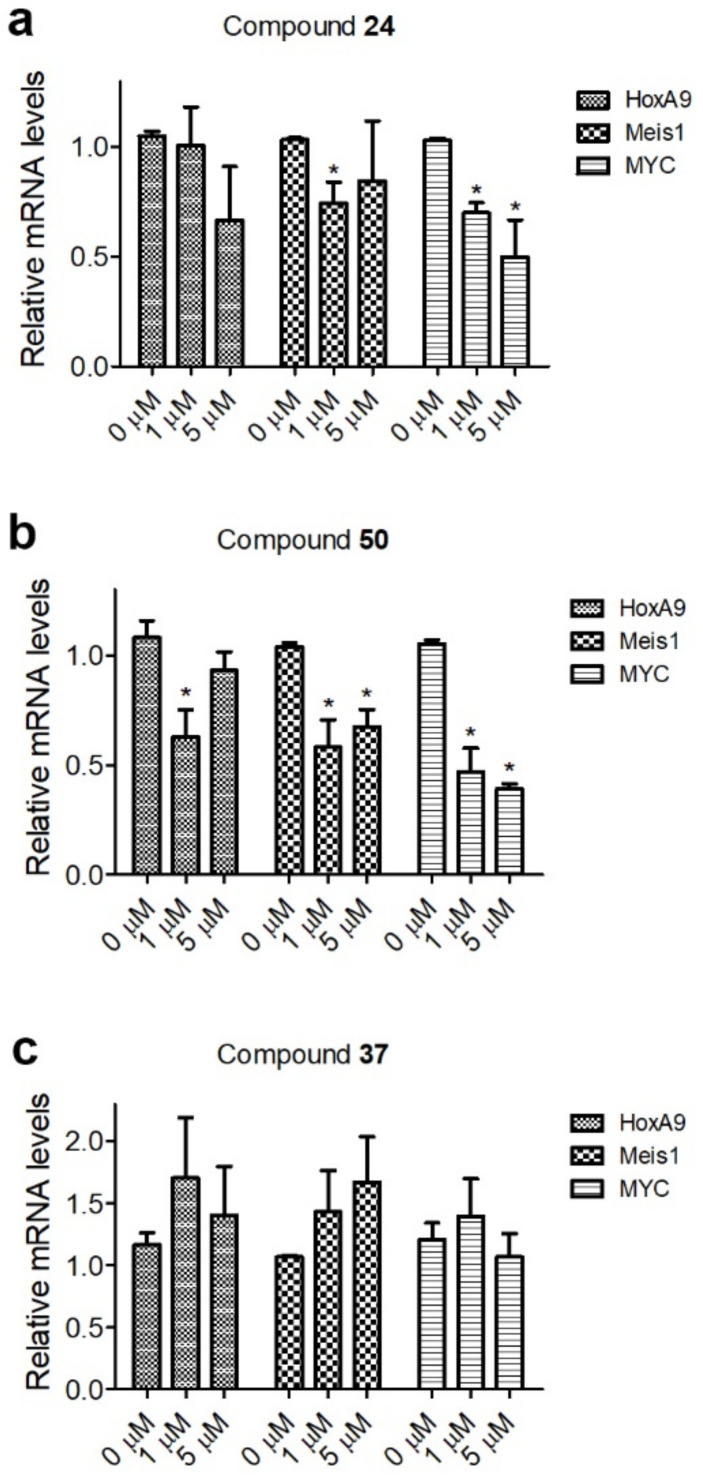
RT-qPCR results of treatment of Molm-13 cells with compounds **24** (**a**), **50** (**b**) or **37** (**c**) at the designated concentrations for 4 days, showing **24** and **50**, but not inactive compound **37**, inhibited expression of representative MLL target genes HoxA9, Meis1 and Myc at 1 and 5 µM in Molm-13 cells. (* *p*   <  0.05).

The major function of the SEC is to promote transcription elongation for its bound genes [20,21,22]. Because of the high affinity of the PPIs between AF9/ENL and AF4, onco-MLL (e.g., MLL-AF4 or MLL-AF9/-ENL) recruits SEC to MLL target genes, facilitates their expression, and eventually causes leukemia initiation [27,28]. Next, we investigated whether selected inhibitors of these PPIs can inhibit the expression of HoxA9, Meis1 and Myc, which are MLL target genes and their high expression is characteristic of MLL-r leukemia. In addition, high expression of Myc is also observed in a broad range of AMLs and other cancers and is considered a driving factor for oncogenesis [38,39,40,41]. To this end, RNAs from the control and compound-treated Molm-13 cells (harboring MLL-AF9) were extracted and subjected to quantitative PCR. As shown in Figure 2, potent inhibitors **24** and **50** were able to reduce the expression of HoxA9, Meis1 and Myc. However, inactive compound **37** did not significantly affect the expression of these MLL target genes.

### 2.7. Antitumor Activity

The antitumor activity of selected compounds was evaluated against the proliferation of a panel of MLL-r leukemia and other cancer cells. The results are shown in Table 7. MV4;11 and Molm-13 leukemia cells contain MLL-AF4 and MLL-AF9 fusion oncogenes, respectively. NB4 and HL60 are AML cells without MLL translocation. Hela (cervical cancer) is a solid tumor cell line. Indole-containing compounds **2**, **12** and **15** were found to exhibit modest to no activities against proliferation of MLL-r leukemia MV4;11 and Molm-13 cells, despite their strong inhibitory activities against the AF9-DOT1L interaction (IC_50_ = 2.9–4.5 μM) (Figure S2). They did not significantly affect the growth of other cancerous cells. Presumably due to improved cell permeability or uptake, benzothiophene-containing inhibitor **24** (IC_50_ = 1.6 μM) showed more potent activities against proliferation of MLL-r leukemia MV4;11 and Molm-13 and AML NB4 and HL60 cells with EC_50_ values of 9.6, 4.7, 5.3 and 10 μM. Benzothiophene compound **25** had generally reduced antitumor activities with EC_50_s of 8.7–26 μM, in line with its reduced biochemical activity (IC_50_ = 5.3 μM). Another benzothiophene inhibitor **50** (IC_50_ = 2.5 μM) exhibited comparable antitumor activities (EC_50_ = 5.1–9.4 μM) to compound **24**. The anti-proliferation activity of these compounds against other AML cells (i.e., NB4 and HL60) might be due to their inhibition of SEC-mediated gene expression (e.g., Myc), as observed in our previous studies [26]. In addition, these three compounds were found to have weak to no activities against the proliferation of Hela cells, showing good selectivity. Benzofuran compound **57** (IC_50_ = 7.2 μM) moderately inhibited the proliferation of MLL-r leukemia and AML cells with EC_50_s of 9.7–21 μM. It did not affect the growth of Hela cells. Moreover, inactive indole, benzothiophene and benzofuran compounds **22**, **37** and **72** were included in this assay as negative controls. None of these three compounds inhibited tumor cell proliferation, largely excluding possible off-target effects for these series of compounds.

## 3. Materials and Methods

All chemicals for synthesis were purchased from Aldrich (Milwaukee, WI, USA) or Alfa Aesar (Ward Hill, MA, USA). Unless otherwise stated, all solvents and reagents were used as received. All reactions were performed using a Teflon-coated magnetic stir bar at the indicated temperature and were conducted under an inert atmosphere when stated. The identity of the synthesized compounds was characterized by ^1^H and ^13^C NMR on a Varian (Palo Alto, CA, USA) 400-MR spectrometer and mass spectrometer (Shimadzu LCMS-2020, Shimadzu Kyoto, Japan). Chemical shifts were reported in parts per million (ppm, δ) downfield from tetramethylsilane. Proton coupling patterns are described as singlet (s), doublet (d), triplet (t), quartet (q), multiplet (m), and broad (br). The identity of the potent inhibitors was confirmed with high resolution mass spectra (HRMS) using an Agilent 6550 iFunnel quadrupole-time-of-flight (Q-TOF) mass spectrometer (Agilent Technologies, Santa Clara, CA, USA) with electrospray ionization (ESI). The purities of the final compounds were determined to be >95% with a Shimadzu Prominence HPLC (Shimadzu, Tokyo, Japan) using a Zorbax C18 (or C8) column (4.6 × 250 mm) (Agilent Technologies, Santa Clara, CA, USA) monitored by UV at 254 nm.

### 3.1. Chemical Synthesis

Synthesis of compounds **1**, **5**, **6**, **7**, **11**, **14**, **15**, **16**, **17**, **18**, **19**, **21** and **22** was reported in our previous publications [42].

#### 3.1.1. General Synthetic Procedure-A for Compounds **3**, **4**, **8**–**10** and **20**

To a solution of 5- or 6-bromoindole-2-carboxylic ester **78** (2.0 g, 7.46 mmol) in THF/H_2_O (10/10 mL), sodium hydroxide (0.90 g, 22.4 mmol) was added slowly. The resulting mixture was stirred and heated at 50 °C for 5 h. After cooling down to room temperature, the solution was removed in a vacuum. The resulting residues were diluted with H_2_O (10 mL) and then acidified with 3 N hydrochloride acid solution to give a white precipitate, which was filtered, washed with cold H_2_O and dried under a vacuum overnight to give the corresponding acid as a white solid, which was used directly for the next step.

The reaction mixture containing crude indole-2-carboxylic acid (1.5 g, 6.25 mmol), 4-aminomethyl-1-Boc-piperidine (1.47 g, 6. 88 mmol), or 4-amino-1-Boc-piperidine (1.38 g, 6.89 mmol), HATU (2. 85 g, 7.5 mmol) and *N*, *N*-diisopropylethylamine (1.84 mL, 12.5 mmol) in DMF (20 mL) was stirred for 12 h at room temperature before quenched with H_2_O (30 mL). The crude product was extracted with ethyl acetate (3 × 30 mL) and the resulting organic phase was washed with brine (3 × 10 mL) and dried over Na_2_SO_4_. Up on removal of the organic solvents, it was purified with column chromatography (silica gel, hexanes: ethyl acetate from 1.5:1 to 1:1), to give compound **79** in a yield of 62–85%.

The reaction mixture containing indole-2-carboxamide **79** (0.2 mmol), aryl boronic acid or aryl-4,4,5,5-tetramethyl-1,3,2-dioxaborolane (0.24 mmol), tetrakis(triphenylphosphine)palladium (11.6 mg, 0.01 mmol), and sodium carbonate (42 mg, 0.4 mmol) in *p*-dioxane/H_2_O (5/1, *v*/*v*, 6 mL) was heated at 100 °C for 16 h. Upon cooling, it was diluted with brine (5 mL), and the crude product was extracted with ethyl acetate (3 × 15 mL). The organic layers were washed with brine (3 × 10 mL), dried over Na_2_SO_4_, and concentrated. The residue was purified with column chromatography (silica gel, hexanes: ethyl acetate from 8:1 to 1:1) to give the corresponding Suzuki coupling product (58–83% yield), which was deprotected in a solution of dichloromethane (3 mL) containing 4N HCl in *p*-dioxane (0.2 mL) at 0 °C to room temperature for 6 h. Upon filtration, the powder was washed with cold water to give the final compounds **3**, **4**, **8**, **9**, **10** and **20** as a hydrochloric salt (~100% yield).

##### 6-(4-(1-Pyrrolinylphenyl)-N-(piperidin-4-ylmethyl)-1H-indole-2-carboxamide hydrochloride (**3**)

^1^H NMR (400 MHz, DMSO-*d*_6_) *δ* 11.66 (s, 1H), 9.02 (s, 1H), 8.71 (s, 2H), 7.64 (d, *J* = 8.3 Hz, 1H), 7.61–7.54 (m, 3H), 7.31 (d, *J* = 8.3 Hz, 1H), 7.15 (s, 1H), 7.00 (s, 2H), 3.39 (s, 4H), 3.29–3.15 (m, 4H), 2.82 (dd, *J* = 21.3, 10.4 Hz, 2H), 2.03 (s, 4H), 1.83 (d, *J* = 12.3 Hz, 3H), 1.41 (dd, *J* = 24.3, 12.7 Hz, 2H). ^13^C NMR (100 MHz, DMSO-*d*_6_) *δ* 161.6, 145.1, 137.6, 136.0, 132.5, 127.9, 126.3, 122.2, 119.4, 115.6, 109.4, 103.1, 50.6, 43.92, 43.2, 34.2, 26.7, 24.9. MS (ESI): [M + H]^+^ 403.2.

##### 6-(4-Cyclohexylphenyl)-N-(piperidin-4-ylmethyl)-1H-indole-2-carboxamide hydrochloride (**4**)

^1^H NMR (400 MHz, DMSO-*d*_6_) *δ* 11.72 (s, 1H), 9.05 (d, *J* = 8.8 Hz, 1H), 8.82–8.68 (m, 2H), 7.66 (d, *J* = 8.4 Hz, 1H), 7.62 (s, 1H), 7.54 (d, *J* = 8.0 Hz, 2H), 7.32 (d, *J* = 8.8 Hz, 1H), 7.29 (d, *J* = 8.0 Hz, 2H), 7.18 (s, 1H), 3.23 (dd, *J* = 14.8, 9.2 Hz, 4H), 2.82 (q, *J* = 11.2 Hz, 2H), 1.90–1.66 (m, 9H), 1.49–1.31 (m, 7H). ^13^C NMR (100 MHz, DMSO-*d*_6_) *δ* 161.1, 146.2, 138.8, 137.1, 135.7, 132.3, 127.2, 126.6, 126.3, 121.8, 119.2, 109.8, 102.6, 43.4, 42.8, 34.0, 33.8, 26.4, 26.3, 25.6, 24.7. MS (ESI): [M + H]^+^ 416.3.

##### 6-(4-Acetophenyl)-N-(piperidin-4-ylmethyl)-1H-indole-2-carboxamide hydrochloride (**8**)

^1^H NMR (400 MHz, DMSO-*d*_6_) *δ* 11.82 (s, 1H), 8.98 (d, *J* = 10.0 Hz, 1H), 8.76 (t, *J* = 5.6 Hz, 1H), 8.68 (d, *J* = 10.0 Hz, 1H), 8.04 (d, *J* = 8.4 Hz, 2H), 7.81 (d, *J* = 8.4 Hz, 2H), 7.73 (d, *J* = 8.8 Hz, 2H), 7.43 (dd, *J* = 8.4, 1.6 Hz, 1H), 7.20 (d, *J* = 1.6 Hz, 1H), 3.23 (dd, *J* = 15.6, 9.6 Hz, 4H), 2.83 (dd, *J* = 22.8, 11.6 Hz, 2H), 2.61 (s, 3H), 1.84 (d, *J* = 12.0 Hz, 3H), 1.42 (dd, *J* = 23.2, 11.2 Hz, 2H). ^13^C NMR (100 MHz, DMSO-*d*_6_) *δ* 197.5, 161.0, 145.7, 136.9, 135.1, 134.2, 133.0, 129.0, 127.2, 126.8, 122.2, 119.3, 110.6, 102.7, 43.6, 42.8, 33.8, 26.8, 26.3. MS (ESI): [M+H]^+^ 375.2.

##### 6-(3-(tert-Butyl)phenyl)-N-(piperidin-4-ylmethyl)-1H-indole-2-carboxamide hydrochloride (**9**)

^1^H NMR (400 MHz, DMSO-*d*_6_) *δ* 11.69 (s, 1H), 9.05 (s, 1H), 8.74 (s, 2H), 7.68 (d, *J* = 8.4 Hz, 1H), 7.63 (d, *J* = 11.6 Hz, 2H), 7.46–7.31 (m, 4H), 7.19 (s, 1H), 3.30–3.18 (m, 4H), 2.83 (dd, *J* = 23.2, 12.0 Hz, 2H), 1.84 (d, *J* = 12.0 Hz, 3H), 1.42 (d, *J* = 12.8 Hz, 2H), 1.34 (s, 9H). ^13^C NMR (100 MHz, DMSO-*d*_6_) *δ* 161.1, 151.2, 141.1, 137.0, 136.3, 132.4, 128.6, 126.4, 124.1, 123.8, 123.6, 121.9, 119.5, 110.2, 102.6, 43.5, 42.8, 34.5, 33.8, 31.2, 26.3. MS (ESI): [M + H]^+^ 389.2.

##### 6-(3-Cyanophenyl)-N-(piperidin-4-ylmethyl)-1H-indole-2-carboxamide hydrochloride (**10**)

^1^H NMR (400 MHz, DMSO-*d*_6_) *δ* 11.87 (s, 1H), 9.08 (d, *J* = 9.6 Hz, 1H), 8.80 (t, *J* = 5.8 Hz, 2H), 8.11 (s, 1H), 8.00 (d, *J* = 7.6 Hz, 1H), 7.79 (d, *J* = 7.6 Hz, 1H), 7.76–7.63 (m, 3H), 7.41 (d, *J* = 8.4 Hz, 1H), 7.22 (s, 1H), 3.23 (dd, *J* = 14.0, 8.8 Hz, 4H), 2.83 (q, *J* = 11.6 Hz, 2H), 1.84 (d, *J* = 12.8 Hz, 3H), 1.43 (q, *J* = 11.6 Hz, 2H). ^13^C NMR (100 MHz, DMSO-*d*_6_) *δ* 161.0, 142.4, 136.9, 133.4, 133.0, 131.6, 130.4, 130.2, 130.2, 127.1, 122.2, 119.2, 118.9, 112.0, 110.5, 102.7, 43.5, 42.8, 33.8, 26.3. MS (ESI): [M + H]^+^ 358.2.

##### 5-(3-Fluoro-4-cyanophenyl)-N-(piperidin-4-ylmethyl)-1H-indole-2-carboxamide hydrochloride (**20**)

^1^H NMR (400 MHz, DMSO-*d*_6_) *δ* 11.82 (s, 1H), 8.79–8.68 (m, 2H), 8.42 (dt, *J* = 10.8, 8.4 Hz, 1H), 8.12 (s, 1H), 7.96 (t, *J* = 7.6 Hz, 1H), 7.90 (dd, *J* = 11.2, 1.2 Hz, 1H), 7.78 (dd, *J* = 8.4, 1.6 Hz, 1H), 7.64 (dd, *J* = 8.8, 1.6 Hz, 1H), 7.53 (d, *J* = 8.8 Hz, 1H), 7.25 (d, *J* = 1.2 Hz, 1H), 3.28–3.20 (m, 4H), 2.91–2.79 (m, 2H), 1.84 (d, *J* = 12.0 Hz, 3H), 1.38 (q, *J* = 12.0 Hz, 2H). MS (ESI): [M + H]^+^ 377.2.

#### 3.1.2. Synthetic Methods for Compounds **2**, **12**, **13** and **23**

To a solution of 5- or 6-substituted indole **80** (3 mmol) in DMF (10 mL) was slowly added to trifluoroacetyl anhydride (0.61 mL, 4.5 mmol) at 0 °C. The resulting mixture was stirred at room temperature for 2 h before quenched with H_2_O (10 mL) to give a white powder, which was filtered and washed with cold water to give compound **81**, which was stirred in a 20% NaOH solution (20 mL) at 50 °C for 2 days. The reaction was slowly acidified with 3 N HCl solution to give a white powder, which was filtered and washed with cold water to give the corresponding indole-3-carboxylic acid, which was directly used without further purification as the starting compound for the general synthetic procedure-A (described above) to give the final compounds **2**, **12**, **13** and **23**.

##### 6-(4-(Piperidin-1-yl)phenyl)-N-(piperidin-4-ylmethyl)-1H-indole-3-carboxamide hydrochloride (**2**)

^1^H NMR (400 MHz, DMSO-*d*_6_) *δ* 11.77 (s, 1H), 8.93 (d, *J* = 10.6 Hz, 1H), 8.64 (d, *J* = 8.6 Hz, 1H), 8.18 (d, *J* = 8.5 Hz, 1H), 8.08 (dd, *J* = 25.9, 12.8 Hz, 2H), 7.92 (d, *J* = 7.8 Hz, 2H), 7.84 (d, *J* = 8.5 Hz, 2H), 7.69 (s, 1H), 7.42 (d, *J* = 8.4 Hz, 1H), 3.22 (d, *J* = 12.1 Hz, 2H), 3.18–3.07 (m, 2H), 2.79 (dd, *J* = 23.1, 11.7 Hz, 3H), 1.80 (d, *J* = 12.2 Hz, 4H), 1.48–1.24 (m, 3H). ^13^C NMR (100 MHz, DMSO-*d*_6_) *δ* 165.0, 142.7, 142.3, 137.1, 132.9, 129.4, 128.4, 126.6, 122.6, 122.0, 120.1, 111.0, 110.5, 66.8, 55.4, 43.7, 43.2, 34.6, 34.4, 26.8, 23.3, 21.3. HRMS (ESI^+^) calcd for C_26_H_32_N_4_O [M + H]^+^, 417.2654; found, 417.2650.

##### 6-(4-(Pyrrolidin-1-yl)phenyl)-N-(piperidin-4-ylmethyl)-1H-indole-3-carboxamide hydrochloride (**12**)

^1^H NMR (400 MHz, DMSO-*d*_6_) *δ* 11.63 (s, 1H), 8.96 (d, *J* = 10.7 Hz, 1H), 8.66 (d, *J* = 10.1 Hz, 1H), 8.19–7.88 (m, 3H), 7.69–7.46 (m, 3H), 7.34 (d, *J* = 8.2 Hz, 1H), 7.02 (d, *J* = 36.4 Hz, 2H), 3.53 (s, 2H), 3.36 (s, 3H), 3.22 (d, *J* = 11.9 Hz, 2H), 3.14 (s, 2H), 2.79 (dd, *J* = 22.3, 11.9 Hz, 2H), 2.00 (s, 3H), 1.80 (d, *J* = 12.2 Hz, 3H), 1.37 (dd, *J* = 23.6, 11.7 Hz, 2H). ^13^C NMR (100 MHz, DMSO-*d*_6_) *δ* 165.1, 145.0, 137.3, 134.5, 128.6, 128.1, 127.9, 125.4, 121.7, 119.6, 115.6, 110.9, 109.1, 66.8, 43.6, 43.3, 34.4, 26.8, 24.9. HRMS (ESI^+^) calcd for C_25_H_30_N_4_O [M + H]^+^, 403.2498; found, 403.2480.

##### 6-(4-(Morpholinophenyl)-N-(piperidin-4-ylmethyl)-1H-indole-3-carboxamide hydrochloride (**13**)

^1^H NMR (400 MHz, DMSO-*d*_6_) *δ* 11.62 (s, 1H), 8.86 (d, *J* = 9.9 Hz, 1H), 8.55 (d, *J* = 11.1 Hz, 1H), 8.13 (d, *J* = 8.4 Hz, 1H), 8.04 (d, *J* = 2.8 Hz, 2H), 7.68–7.52 (m, 3H), 7.36 (d, *J* = 8.4 Hz, 1H), 7.21 (d, *J* = 7.9 Hz, 2H), 3.99–3.75 (m, 5H), 3.32–3.19 (m, 5H), 3.15 (t, *J* = 5.1 Hz, 2H), 2.81 (dd, *J* = 23.0, 11.1 Hz, 2H), 1.81 (d, *J* = 11.2 Hz, 3H), 1.48–1.29 (m, 2H). ^13^C NMR (100 MHz, DMSO-*d*_6_) *δ* 165.1, 137.3, 134.2, 128.7, 127.8, 125.6, 121.8, 119.8, 117.2, 111.0, 110.0, 109.4, 66.0, 50.0, 43.6, 43.3, 34.4, 26.8. MS (ESI): [M + H]^+^ 419.2.

##### 5-(4-(Piperidin-1-yl)phenyl)-N-(piperidin-4-ylmethyl)-1H-indole-3-carboxamide hydrochloride (**23**)

^1^H NMR (400 MHz, DMSO-*d*_6_) *δ* 11.75 (s, 1H), 8.91 (s, 1H), 8.64 (s, 1H), 8.41 (s, 1H), 8.19–8.04 (m, 2H), 7.92 (d, *J* = 7.5 Hz, 2H), 7.80 (d, *J* = 8.6 Hz, 2H), 7.48 (dt, *J* = 8.6, 4.9 Hz, 2H), 3.55 (d, *J* = 11.6 Hz, 10H), 3.22 (d, *J* = 11.5 Hz, 2H), 3.16 (t, *J* = 5.7 Hz, 2H), 2.80 (dd, *J* = 22.4, 11.4 Hz, 2H), 1.80 (d, *J* = 11.8 Hz, 3H), 1.38 (dd, *J* = 23.4, 11.3 Hz, 2H). ^13^C NMR (100 MHz, DMSO-*d*_6_) *δ* 165.0, 136.4, 131.6, 129.2, 128.4, 127.1, 122.4, 121.7, 119.8, 112.9, 111.3, 66.8, 55.4, 43.7, 43.3, 34.4, 26.8, 23.4. MS (ESI): [M + H]^+^ 417.3.

#### 3.1.3. Synthetic Methods for Compounds **24**–**28**, **37**–**50**, **53**, **54**, **58**–**62**, and **67**–**73**

General synthetic procedure-A was used for the synthesis of compounds **24**–**28**, **37**–**50, 53**, **54**, **58**–**62** and **67**–**73**, starting from a Br- or I-substituted benzothiophene- or benzofuran-2-carboxylic acid **83**.

##### 6-(4-(Piperidin-1-yl)phenyl)-N-(piperidin-4-ylmethyl)-benzo[b]thiophene-2-carboxamide hydrochloride (**24**)

^1^H NMR (500 MHz, CD_3_OD) *δ* 8.23 (s, 1H), 8.05 (s, 1H), 8.02 (d, *J* = 8.4 Hz, 1H), 7.97–7.93 (m, 2H), 7.84–7.80 (m, 2H), 7.74 (dd, *J* = 8.4, 1.6 Hz, 1H), 3.72–3.66 (m, 4H), 3.46–3.41 (m, 2H), 3.39–3.36 (m, 2H), 3.05–2.97 (m, 2H), 2.17–2.08 (m, 4H), 2.05–1.98 (m, 3H), 1.88–1.80 (m, 2H), 1.60–1.50 (m, 2H). ^13^C NMR (126 MHz, CD_3_OD) *δ* 164.9, 143.7, 143.2, 143.1, 140.9, 140.5, 138. 7, 130.3, 126.9, 126.3, 125.5, 122.8, 122.0, 58.3, 45.5, 44.9, 35.4, 27.7, 24.8, 22.2. HRMS (ESI^+^) calcd for C_26_H_31_N_3_O_3_S [M + H]^+^, 434.2266; found, 434.2261.

##### 6-(4-(Pyrrolindin-1-yl)phenyl)-N-(piperidin-4-ylmethyl)-benzo[b]thiophene-2-carboxamide hydrochloride (**25**)

^1^H NMR (500 MHz, DMSO-*d*_6_) *δ* 9.06 (br, 1H), 8.98–8.90 (m, 1H), 8.76 (br, 1H), 8.23 (s, 1H), 8.13 (s, 1H), 7.93 (d, *J* = 8.4 Hz, 1H), 7.74–7.65 (m, 3H), 6.89–6.80 (m, 2H), 3.39–3.31 (m, 4H), 3.28–3.21 (m, 2H), 3.19 (t, *J* = 6.0 Hz, 2H), 2.88–2.78 (m, 2H), 2.06–1.95 (m, 4H), 1.88–1.78 (m, 3H), 1.48–1.35 (m, 2H). ^13^C NMR (126 MHz, DMSO-*d*_6_) *δ* 161.6, 141.2, 139.3, 138.2, 137.3, 127.6, 125.2, 124.5, 123.2, 118.9, 43.9, 42.6, 33.6, 30.6, 26.2, 24.6. HRMS (ESI^+^) calcd for C_25_H_29_N_3_OS [M + H]^+^, 420.2110; found, 420.2096.

##### 6-(4-(Morpholino)phenyl)-N-(piperidin-4-ylmethyl)-benzo[b]thiophene-2-carboxamide hydrochloride (**26**)

^1^H NMR (400 MHz, DMSO-*d*_6_) *δ* 8.98–8.88 (m, 2H), 8.68–8.55 (m, 1H), 8.26 (s, 1H), 8.12 (s, 1H), 7.94 (d, *J* = 8.4 Hz, 1H), 7.75–7.65 (m, 3H), 7.15 (d, *J* = 8.2 Hz, 2H), 3.83–3.75 (m, 4H), 3.28–3.16 (m, 8H), 2.89–2.75 (m, 2H), 1.88–1.75 (m, 3H), 1.45–1.30 (m, 2H). ^13^C NMR (100 MHz, DMSO-*d*_6_) *δ* 161.6, 141.1, 139.7, 137.9, 137.7, 127.6, 125.3, 124.5, 123.5, 119.5, 116.0, 65.7, 48.7, 44.0, 42.8, 33.6, 26.2. MS (ESI): [M + H]^+^ 436.2.

##### 6-(4-(Diethylamino)phenyl)-N-(piperidin-4-ylmethyl)-benzo[b]thiophene-2-carboxamide hydrochloride (**27**)

^1^H NMR (400 MHz, DMSO-*d*_6_) *δ* 10.60–10.45 (m, 1H), 8.97 (t, *J* = 5.6 Hz, 1H), 8.87 (br, 1H), 8.58 (br, 1H), 8.39 (s, 1H), 8.16 (s, 1H), 8.02 (d, *J* = 8.4 Hz, 1H), 7.87 (d, *J* = 8.2 Hz, 2H), 7.83–7.71 (m, 3H), 3.29–3.15 (m, 4H), 3.12–2.99 (m, 4H), 2.92–2.76 (m, 2H), 1.92–1.75 (m, 3H), 1.44–1.34 (m, 2H), 1.26 (t, *J* = 7.2 Hz, 6H). ^13^C NMR (100 MHz, DMSO-*d*_6_) *δ* 161.6, 141.1, 140.7, 140.4, 138.7, 137.2, 131.8, 129.7, 127.3, 125.6, 124.4, 124.0, 120.8, 45.7, 44.0, 42.8, 33.7, 26.3, 8.3. MS (ESI): [M + H]^+^ 422.2.

##### 6-(4-(Piperidin-1-ylmethyl)phenyl)-N-(piperidin-4-ylmethyl)-benzo[b]thiophene-2-carboxamide hydrochloride (**28**)

^1^H NMR (400 MHz, DMSO-*d*_6_) *δ* 10.69 (br, 1H), 9.05–8.93 (m, 2H), 8.73–8.60 (m, 1H), 8.38 (s, 1H), 8.17 (s, 1H), 8.02 (d, *J* = 8.4 Hz, 1H), 7.86 (d, *J* = 8.1 Hz, 2H), 7.78 (d, *J* = 8.4 Hz, 1H), 7.72 (d, *J* = 8.1 Hz, 2H), 4.35–4.23 (m, 2H), 3.32–3.14 (m, 6H), 2.91–2.73 (m, 4H), 1.90–1.62 (m, 8H), 1.45–1.27 (m, 3H). ^13^C NMR (100 MHz, DMSO-*d*_6_) *δ* 161.6, 141.1, 140.7, 140.5, 138.7, 137.2, 132.1, 129.2, 127.2, 125.6, 124.5, 124.1, 120.8, 58.4, 51.6, 44.0, 42.8, 33.7, 26.3, 22.1, 21.4. MS (ESI): [M + H]^+^ 448.2.

##### 6-(4-(Carbamoyl)phenyl)-N-(piperidin-4-ylmethyl)-benzo[b]thiophene-2-carboxamide hydrochloride (**37**)

^1^H NMR (400 MHz, DMSO-*d*_6_) *δ* 9.05–8.96 (m, 2H), 8.75–8.65 (m, 1H), 8.42 (s, 1H), 8.18 (s, 1H), 8.09–7.95 (m, 4H), 7.86 (d, *J* = 8.4 Hz, 2H), 7.80 (dd, *J* = 8.4, 1.4 Hz, 1H), 7.41 (s, 1H), 3.30–3.16 (m, 4H), 2.89–2.75 (m, 2H), 1.95–1.80 (m, 3H), 1.48–1.32 (m, 2H). ^13^C NMR (100 MHz, DMSO-*d*_6_) *δ* 167.4, 161.6, 142.2, 141.1, 140.8, 138.8, 137.1, 133.2, 128.2, 126.7, 125.6, 124.5, 124.1, 121.0, 44.0, 42.8, 33.7, 26.3. MS (ESI): [M + H]^+^ 394.2.

##### 6-(4-(Acetamido)phenyl)-N-(piperidin-4-ylmethyl)-benzo[b]thiophene-2-carboxamide hydrochloride (**38**)

^1^H NMR (400 MHz, DMSO-*d*_6_) *δ* 10.12 (s, 1H), 8.91 (t, *J* = 5.3 Hz, 1H), 8.86–8.75 (m, 1H), 8.58–8.42 (m, 1H), 8.28 (s, 1H), 8.12 (s, 1H), 7.97 (d, *J* = 8.4 Hz, 1H), 7.70–7.50 (m, 5H), 3.30–3.14 (m, 4H), 2.90–2.74 (m, 2H), 2.07 (s, 3H), 1.89–1.75 (m, 3H), 1.45–1.30 (m, 2H). ^13^C NMR (100 MHz, DMSO-*d*_6_) *δ* 168.4, 161.7, 141.1, 140.0, 139.1, 138.0, 134.0, 127.2, 126.3, 125.4, 124.5, 123.7, 119.9, 119.3, 44.0, 42.8, 33.7, 26.3, 24.1. MS (ESI): [M + H]^+^ 408.2.

##### 6-(4-(Hydroxylmethyl)phenyl)-N-(piperidin-4-ylmethyl)-benzo[b]thiophene-2-carboxamide hydrochloride (**39**)

^1^H NMR (400 MHz, DMSO-*d*_6_) *δ* 8.95–8.88 (m, 1H), 8.80–8.68 (m, 1H), 8.49–8.37 (m, 1H), 8.32 (s, 1H), 8.12 (s, 1H), 8.03–7.96 (m, 1H), 7.80–7.70 (m, 3H), 7.42 (d, *J* = 7.6 Hz, 2H), 5.25 (br, 1H), 4.55 (s, 2H), 3.24–3.13 (m, 4H), 2.89–2.75 (m, 3H), 1.89–1.78 (m, 3H), 1.45–1.30 (m, 2H). ^13^C NMR (100 MHz, DMSO-*d*_6_) *δ* 161.7, 142.2, 141.1, 140.2, 138.2, 138.1, 138.0, 127.1, 126.7, 125.5, 124.4, 124.0, 120.4, 62.6, 44.0, 42.9, 33.7, 26.3. MS (ESI): [M + H]^+^ 381.2.

##### 6-(4-(Tert-butyl)phenyl)-N-(piperidin-4-ylmethyl)-benzo[b]thiophene-2-carboxamide hydrochloride (**40**)

^1^H NMR (400 MHz, DMSO-*d*_6_) *δ* 8.95–8.85 (m, 1H), 8.73 (br, 1H), 8.48 (br, 1H), 8.28 (s, 1H), 8.11 (s, 1H), 7.97 (d, *J* = 8.4 Hz, 1H), 7.75–7.65 (m, 3H), 7.48 (d, *J* = 8.4 Hz, 2H), 3.27–3.13 (m, 4H), 2.82 (t, *J* = 12.1 Hz, 2H), 1.89–1.75 (m, 3H), 1.40–1.30 (m, 2H), 1.30 (s, 9H). ^13^C NMR (100 MHz, DMSO-*d*_6_) *δ* 161.7, 150.2, 141.1, 140.1, 138.2, 138.1, 136.8, 126.7, 125.8, 125.5, 124.5, 124.0, 120.3, 44.0, 42.8, 34.3, 33.7, 31.1, 26.3. MS (ESI): [M + H]^+^ 407.2.

##### 6-(4-(Cyanomethyl)phenyl)-N-(piperidin-4-ylmethyl)-benzo[b]thiophene-2-carboxamide hydrochloride (**41**)

^1^H NMR (400 MHz, DMSO-*d*_6_) *δ* 8.98–8.85 (m, 2H), 8.65–8.55 (m, 1H), 8.35–8.28 (m, 1H), 8.14 (d, *J* = 4.9 Hz, 1H), 8.03–7.94 (m, 1H), 7.80 (d, *J* = 8.3 Hz, 1H), 7.76–7.68 (m, 2H), 7.46 (d, *J* = 8.3 Hz, 1H), 7.37 (d, *J* = 8.3 Hz, 1H), 4.10 (s, 1H), 3.42 (s, 1H), 3.29–3.16 (m, 4H), 2.88–2.75 (m, 2H), 1.88–1.75 (m, 3H), 1.46–1.29 (m, 2H). MS (ESI): [M + H]^+^ 390.2.

##### 6-(4-Cyano-3-fluorophenyl)-N-(piperidin-4-ylmethyl)-benzo[b]thiophene-2-carboxamide hydrochloride (**42**)

^1^H NMR (400 MHz, DMSO-*d*_6_) *δ* 9.05–8.96 (m, 1H), 8.88–8.80 (m, 1H), 8.60–8.47 (m, 2H), 8.17 (s, 1H), 8.11–7.97 (m, 3H), 7.89–7.84 (m, 2H), 3.28–3.15 (m, 4H), 2.90–2.78 (m, 2H), 1.88–1.76 (m, 3H), 1.45–1.30 (m, 2H). ^13^C NMR (100 MHz, DMSO-*d*_6_) *δ* 162.9 (d, *J* = 254.7 Hz), 161.4, 147.2 (d, *J* = 8.5 Hz), 141.8, 140.9, 139.7, 134.7 (d, *J* = 1.9 Hz), 134.3, 125.8, 124.4, 124.1, 123.8 (d, *J* = 2.8 Hz), 121.8, 114.6 (d, *J* = 20.7 Hz), 114.1, 98.7 (d, *J* = 15.3 Hz), 44.0, 42.8, 33.6, 26.3. MS (ESI): [M + H]^+^ 394.1.

##### 6-(4-(Methylsulfonyl)phenyl)-N-(piperidin-4-ylmethyl)-benzo[b]thiophene-2-carboxamide hydrochloride (**43**)

^1^H NMR (400 MHz, DMSO-*d*_6_) *δ* 8.96 (t, *J* = 6.0 Hz, 1H), 8.78–8.70 (m, 1H), 8.50–8.35 (m, 2H), 8.16 (s, 1H), 8.09–7.99 (m, 5H), 7.83 (dd, *J* = 8.4, 1.7 Hz, 1H), 3.28–3.25 (m, 5H), 3.20 (t, *J* = 6.1 Hz, 2H), 2.88–2.78 (m, 2H), 1.89–1.80 (m, 3H), 1.42–1.30 (m, 2H). ^13^C NMR (150 MHz, DMSO-*d*_6_) *δ* 162.0, 145.1, 141.7, 141.5, 140.2, 139.7, 136.7, 128.4, 128.2, 126.2, 124.9, 124.8, 122.0, 44.5, 44.0, 43.4, 34.1, 26.8. MS (ESI): [M + H]^+^ 429.1.

##### 6-(4-Trifluoromethoxylphenyl)-N-(piperidin-4-ylmethyl)-benzo[b]thiophene-2-carboxamide hydrochloride (**44**)

^1^H NMR (400 MHz, DMSO-*d*_6_) *δ* 9.05–8.95 (m, 2H), 8.75–8.60 (m, 1H), 8.36 (s, 1H), 8.17 (s, 1H), 8.02 (d, *J* = 8.4 Hz, 1H), 7.89 (d, *J* = 8.7 Hz, 2H), 7.75 (d, *J* = 8.4 Hz, 1H), 7.48 (d, *J* = 8.4 Hz, 2H), 3.28–3.10 (m, 4H), 2.89–2.76 (m, 2H), 1.89–1.75 (m, 3H), 1.45–1.32 (m, 2H). ^13^C NMR (100 MHz, DMSO-*d*_6_) *δ* 161.6, 148.0, 141.0, 140.7, 139.0, 138.7, 136.6, 128.9, 125.6, 124.4, 124.1, 121.5, 120.9, 44.0, 42.8, 33.7, 26.3. MS (ESI): [M + H]^+^ 435.1.

##### 6-(4-Methoxylphenyl)-N-(piperidin-4-ylmethyl)-benzo[b]thiophene-2-carboxamide hydrochloride (**45**)

^1^H NMR (400 MHz, DMSO-*d*_6_) *δ* 9.18–9.06 (m, 1H), 9.00 (t, *J* = 5.3 Hz, 1H), 8.88–8.75 (m, 1H), 8.25 (s, 1H), 8.17 (s, 1H), 7.94 (d, *J* = 8.4 Hz, 1H), 7.74–7.65 (m, 3H), 7.03 (d, *J* = 8.7 Hz, 2H), 3.79 (s, 3H), 3.27–3.11 (m, 4H), 2.89–2.72 (m, 2H), 1.89–1.75 (m, 3H), 1.51–1.28 (m, 2H). ^13^C NMR (100 MHz, DMSO-*d*_6_) *δ* 161.7, 159.1, 141.1, 139.9, 137.9, 137.8, 131.9, 128.1, 125.4, 124.6, 123.7, 119.8, 114.5, 55.2, 44.0, 42.7, 33.7, 26.3. MS (ESI): [M + H]^+^ 381.2.

##### 6-(4-Hydroxylphenyl)-N-(piperidin-4-ylmethyl)-benzo[b]thiophene-2-carboxamide hydrochloride (**46**)

^1^H NMR (400 MHz, DMSO-*d*_6_) *δ* 9.61 (s, 1H), 8.92 (t, *J* = 5.1 Hz, 1H), 8.85–8.78 (m, 1H), 8.58–8.46 (m, 1H), 8.24 (s, 1H), 8.12 (s, 1H), 7.98 (d, *J* = 8.5 Hz, 1H), 7.66 (d, *J* = 8.4 Hz, 1H), 7.27 (t, *J* = 7.8 Hz, 1H), 7.16 (d, *J* = 7.8 Hz, 1H), 7.11 (s, 1H), 6.80 (d, *J* = 8.0 Hz, 1H), 3.27–3.18 (m, 4H), 2.88–2.74 (m, 2H), 1.89–1.78 (m, 3H), 1.45–1.30 (m, 2H). ^13^C NMR (100 MHz, DMSO-*d*_6_) *δ* 161.7, 157.9, 141.1, 141.0, 140.3, 138.4, 138.4, 130.0, 125.4, 124.4, 124.1, 120.5, 117.8, 114.7, 113.8, 44.0, 42.8, 33.7, 26.3. MS (ESI): [M + H]^+^ 367.1.

##### 6-(4-Methylphenyl)-N-(piperidin-4-ylmethyl)-benzo[b]thiophene-2-carboxamide hydrochloride (**47**)

^1^H NMR (400 MHz, DMSO-*d*_6_) *δ* 8.99–8.85 (m, 2H), 8.65–8.55 (m, 1H), 8.30 (s, 1H), 8.13 (s, 1H), 7.98 (d, *J* = 8.4 Hz, 1H), 7.72 (dd, *J* = 8.4, 1.6 Hz, 1H), 7.66 (d, *J* = 8.1 Hz, 2H), 7.29 (d, *J* = 8.0 Hz, 2H), 3.29–3.15 (m, 4H), 2.89–2.75 (m, 2H), 2.35 (s, 3H), 1.89–1.75 (m, 3H), 1.45–1.30 (m, 2H). ^13^C NMR (100 MHz, DMSO-*d*_6_) *δ* 161.7, 141.1, 140.1, 138.1, 138.1, 137.1, 136.7, 129.6, 126.8, 125.4, 124.5, 123.9, 120.2, 44.0, 42.8, 33.7, 26.3, 20.7. MS (ESI): [M + H]^+^ 365.2.

##### 6-(4-Chlorophenyl)-N-(piperidin-4-ylmethyl)-benzo[b]thiophene-2-carboxamide hydrochloride (**48**)

^1^H NMR (400 MHz, DMSO-*d*_6_) *δ* 8.96–8.90 (m, 1H), 8.82–8.72 (m, 1H), 8.50–8.40 (m, 1H), 8.35 (s, 1H), 8.13 (s, 1H), 8.01 (d, *J* = 8.5 Hz, 1H), 7.80 (d, *J* = 8.5 Hz, 2H), 7.76–7.72 (m, 1H), 7.54 (d, *J* = 8.5 Hz, 2H), 3.28–3.17 (m, 4H), 2.90–2.74 (m, 2H), 1.88–1.78 (m, 3H), 1.46–1.28 (m, 2H). ^13^C NMR (100 MHz, DMSO-*d*_6_) *δ* 161.6, 141.1, 140.6, 138.6, 138.5, 136.8, 132.6, 129.0, 128.8, 125.6, 124.4, 124.0, 120.7, 44.0, 42.9, 33.7, 26.3. MS (ESI): [M + H]^+^ 385.1.

##### 6-(4-Fluorophenyl)-N-(piperidin-4-ylmethyl)-benzo[b]thiophene-2-carboxamide hydrochloride (**49**)

^1^H NMR (400 MHz, DMSO-*d*_6_) *δ* 9.02–8.89 (m, 2H), 8.63 (br, 1H), 8.31 (s, 1H), 8.15 (s, 1H), 7.99 (d, *J* = 8.4 Hz, 1H), 7.85–7.76 (m, 2H), 7.72 (d, *J* = 8.4 Hz, 1H), 7.35–7.28 (m, 2H), 3.26–3.15 (m, 4H), 2.88–2.75 (m, 2H), 1.89–1.75 (m, 3H), 1.45–1.32 (m, 2H). ^13^C NMR (100 MHz, DMSO-*d*_6_) *δ* 162.0 (d, *J* = 244.0 Hz), 161.6, 141.0, 140.4, 138.3, 137.1, 136.1 (d, *J* = 3.0 Hz), 129.0 (d, *J* = 8.2 Hz), 125.5, 124.4, 124.0, 120.6, 115.8 (d, *J* = 21.4 Hz), 44.0, 42.8, 33.7, 26.3. MS (ESI): [M + H]^+^ 369.1.

##### 5-(4-(Piperidin-1-yl)phenyl)-N-(piperidin-4-ylmethyl)-benzo[b]thiophene-2-carboxamide hydrochloride (**50**)

^1^H NMR (400 MHz, DMSO-*d*_6_) *δ* 9.15–9.02 (m, 2H), 8.85–8.70 (m, 1H), 8.25–8.19 (m, 2H), 8.10 (d, *J* = 8.5 Hz, 1H), 7.99–7.85 (m, 3H), 7.79–7.75 (m, 1H), 7.69 (d, *J* = 9.0 Hz, 1H), 6.89 (d, *J* = 9.0 Hz, 1H), 3.40–3.34 (m, 2H), 3.33–3.13 (m, 6H), 2.89–2.75 (m, 2H), 2.11–1.63 (m, 9H), 1.49–1.40 (m, 2H). ^13^C NMR (100 MHz, DMSO-*d*_6_) *δ* 161.6, 141.0, 139.9, 139.7, 135.9, 134.5, 128.3, 125.2, 125.0, 123.5, 123.0, 122.8, 116.1, 56.3, 44.1, 42.8, 33.7, 26.3, 23.2, 22.9. HRMS (ESI^+^) calcd for C_26_H_31_N_3_O_3_S [M + H]^+^, 434.2266; found, 434.2263.

##### 5-(4-Cyanophenyl)-N-(piperidin-4-ylmethyl)-benzo[b]thiophene-2-carboxamide hydrochloride (**53**)

^1^H NMR (400 MHz, DMSO-*d*_6_) *δ* 9.05 (t, *J* = 5.9 Hz, 1H), 8.99–8.90 (m, 1H), 8.70–8.56 (m, 1H), 8.30 (d, *J* = 1.5 Hz, 1H), 8.20 (s, 1H), 8.14 (d, *J* = 8.5 Hz, 1H), 8.01–7.92 (m, 4H), 7.83 (dd, *J* = 8.5, 1.8 Hz, 1H), 3.27–3.16 (m, 4H), 2.89–2.70 (m, 2H), 1.89–1.70 (m, 3H), 1.46–1.28 (m, 2H). ^13^C NMR (100 MHz, DMSO-*d*_6_) *δ* 161.5, 144.4, 141.2, 140.5, 139.9, 135.3, 132.9, 127.9, 125.2, 125.0, 123.6, 123.6, 118.9, 110.0, 44.1, 42.8, 33.7, 26.3. MS (ESI): [M + H]^+^ 376.1.

##### 5-(4-(Tert-butyl)phenyl)-N-(piperidin-4-ylmethyl)-benzo[b]thiophene-2-carboxamide hydrochloride (**54**)

^1^H NMR (400 MHz, DMSO-*d*_6_) *δ* 8.97 (t, *J* = 5.9 Hz, 1H), 8.90–8.80 (m, 1H), 8.60–8.50 (m, 1H), 8.18–8.11 (m, 2H), 8.07 (d, *J* = 8.5 Hz, 1H), 7.73 (dd, *J* = 8.5, 1.6 Hz, 1H), 7.66 (d, *J* = 8.4 Hz, 2H), 7.50 (d, *J* = 8.4 Hz, 2H), 3.28–3.14 (m, 4H), 2.89–2.75 (m, 2H), 1.91–1.75 (m, 3H), 1.45–1.34 (m, 2H), 1.32 (s, 9H). ^13^C NMR (100 MHz, DMSO-*d*_6_) *δ* 161.6, 150.0, 140.7, 139.9, 139.1, 137.2, 137.1, 126.7, 125.8, 125.2, 125.0, 123.3, 122.6, 44.0, 42.8, 34.3, 33.7, 31.1, 26.3. MS (ESI): [M + H]^+^ 407.2.

##### 6-(4-Morpholinophenyl)-N-(piperidin-4-ylmethyl)-benzofuran-2-carboxamide hydrochloride (**58**)

^1^H NMR (400 MHz, DMSO-*d*_6_) *δ* 9.05–8.95 (m, 1H), 8.87 (t, *J* = 5.8 Hz, 1H), 8.69–8.60 (m, 1H), 7.85–7.73 (m, 2H), 7.67 (d, *J* = 8.4 Hz, 2H), 7.61 (d, *J* = 8.2 Hz, 1H), 7.56 (s, 1H), 7.19–7.01 (m, 2H), 3.85–3.70 (m, 4H), 3.29–3.15 (m, 8H), 2.89–2.72 (m, 2H), 1.89–1.75 (m, 3H), 1.45–1.30 (m, 2H). ^13^C NMR (100 MHz, DMSO-*d*_6_) *δ* 158.3, 155.1, 149.3, 139.1, 127.7, 125.7, 122.9, 122.4, 115.9, 109.4, 108.5, 65.8, 48.6, 43.4, 42.8, 33.6, 26.2. MS (ESI): [M + H]^+^ 420.2.

##### 6-(4-(Piperazin-1-yl)phenyl)-N-(piperidin-4-ylmethyl)-benzofuran-2-carboxamide hydrochloride (**59**)

^1^H NMR (400 MHz, DMSO-*d*_6_) *δ* 9.35–9.25 (m, 2H), 8.99–8.90 (m, 1H), 8.88–8.80 (m, 1H), 8.70–8.58 (m, 1H), 7.85–7.74 (m, 2H), 7.70–7.54 (m, 4H), 7.10 (d, *J* = 8.4 Hz, 2H), 3.44 (d, *J* = 3.6 Hz, 4H), 3.22 (t, *J* = 11.0 Hz, 8H), 2.81 (dd, *J* = 17.8, 6.6 Hz, 2H), 1.83 (dd, *J* = 18.5, 8.7 Hz, 3H), 1.38 (dd, *J* = 20.2, 8.6 Hz, 2H). ^13^C NMR (100 MHz, DMSO-*d*_6_) *δ* 158.3, 155.1, 149.5, 149.3, 139.0, 131.0, 127.7, 125.7, 122.9, 122.4, 116.2, 109.3, 108.6, 45.1, 43.4, 42.7, 42.4, 33.6, 26.2. MS (ESI): [M + H]^+^ 419.2.

##### 6-(4-(4-Methylpiperazin-1-yl)phenyl)-N-(piperidin-4-ylmethyl)-benzofuran-2-carboxamide hydrochloride (**60**)

^1^H NMR (400 MHz, DMSO-*d*_6_) *δ* 10.89 (br, 1H), 8.95–8.80 (m, 2H), 8.65–8.50 (m, 1H), 7.86–7.72 (m, 2H), 7.68–7.54 (m, 4H), 7.11 (d, *J* = 8.4 Hz, 2H), 3.95–3.85 (m, 2H), 3.52–3.45 (m, 2H), 3.27–3.09 (m, 8H), 2.86–2.75 (m, 5H), 1.90–1.75 (m, 3H), 1.42–1.30 (m, 2H). ^13^C NMR (100 MHz, DMSO-*d*_6_) *δ* 158.3, 155.1, 149.3, 149.1, 139.0, 131.0, 127.7, 125.7, 122.9, 122.4, 116.2, 109.3, 108.6, 51.9, 45.2, 43.4, 42.8, 41.9, 33.6, 26.2. MS (ESI): [M + H]^+^ 433.3.

##### 6-(4-Cyclohexylphenyl)-N-(piperidin-4-ylmethyl)-benzofuran-2-carboxamide hydrochloride (**61**)

^1^H NMR (400 MHz, DMSO-*d*_6_) *δ* 8.87 (t, *J* = 5.7 Hz, 1H), 8.78 (br, 1H), 8.47 (br, 1H), 7.90–7.76 (m, 2H), 7.68–7.60 (m, 3H), 7.56 (s, 1H), 7.33 (d, *J* = 8.0 Hz, 2H), 3.29–3.15 (m, 4H), 2.89–2.76 (m, 2H), 2.58–2.50 (m, 1H), 1.89–1.73 (m, 7H), 1.72–1.67 (m, 1H), 1.48–1.18 (m, 7H). ^13^C NMR (100 MHz, DMSO-*d*_6_) *δ* 158.3, 155.0, 149.5, 147.2, 139.4, 137.5, 127.4, 127.1, 126.2, 123.0, 122.9, 109.4, 109.3, 43.5, 42.9, 33.9, 33.7, 26.4, 26.3, 25.6. MS (ESI): [M + H]^+^ 417.2.

##### 6-(4-(Diethylamino)phenyl)-N-(piperidin-4-ylmethyl)-benzofuran-2-carboxamide hydrochloride (**62**)

^1^H NMR (400 MHz, DMSO-*d*_6_) *δ* 10.41 (br, 1H), 8.90 (t, *J* = 5.9 Hz, 1H), 8.88–8.75 (m, 1H), 8.55–8.40 (m, 1H), 7.95 (s, 1H), 7.90–7.79 (m, 3H), 7.74–7.65 (m, 3H), 7.60 (s, 1H), 4.33 (d, *J* = 5.3 Hz, 2H), 3.27–3.16 (m, 6H), 2.85–2.75 (m, 2H), 1.89–1.75 (m, 3H), 1.45–1.35 (m, 2H), 1.29–1.20 (m, 6H). ^13^C NMR (100 MHz, DMSO-*d*_6_) *δ* 158.6, 155.4, 150.2, 141.0, 138.7, 132.2, 130.1, 127.8, 127.3, 123.6, 123.4, 110.1, 109.7, 46.1, 43.9, 43.2, 40.6, 40.4, 40.1, 34.1, 26.7, 8.7. MS (ESI): [M + H]^+^ 406.2.

##### 6-((1,1′-Biphenyl)-4-yl)-N-(piperidin-4-ylmethyl)-benzofuran-2-carboxamide hydrochloride (**67**)

^1^H NMR (400 MHz, DMSO-*d*_6_) *δ* 8.90 (t, *J* = 5.9 Hz, 1H), 8.85–8.75 (m, 1H), 8.55–8.45 (m, 1H), 7.95 (s, 1H), 7.88–7.84 (m, 3H), 7.82–7.75 (m, 2H), 7.74–7.68 (m, 2H), 7.60 (s, 1H), 7.53–7.46 (m, 2H), 7.40–7.35 (m, 1H), 3.28–3.13 (m, 4H), 2.89–2.75 (m, 2H), 1.91–1.70 (m, 3H), 1.44–1.27 (m, 2H). ^13^C NMR (100 MHz, DMSO-*d*_6_) *δ* 158.3, 155.0, 149.7, 139.5, 139.4, 138.8, 138.7, 129.1, 127.7, 127.3, 126.6, 126.6, 123.1, 122.9, 109.5, 109.4, 43.5, 42.8, 33.6, 26.3. MS (ESI): [M + H]^+^ 411.2.

##### 6-((1,1′-Biphenyl)-3-yl)-N-(piperidin-4-ylmethyl)-benzofuran-2-carboxamide hydrochloride (**68**)

^1^H NMR (400 MHz, DMSO-*d*_6_) *δ* 8.95–8.78 (m, 2H), 8.59–8.48 (m, 1H), 8.05–7.95 (m, 2H), 7.86 (d, *J* = 8.2 Hz, 1H), 7.79–7.71 (m, 4H), 7.69–7.65 (m, 1H), 7.62–7.55 (m, 2H), 7.55–7.45 (m, 2H), 7.43–7.35 (m, 1H), 3.29–3.13 (m, 4H), 2.88–2.75 (m, 2H), 1.89–1.75 (m, 3H), 1.45–1.30 (m, 2H). ^13^C NMR (100 MHz, DMSO-*d*_6_) *δ* 158.3, 155.0, 149.7, 141.1, 140.6, 140.1, 139.3, 129.7, 129.0, 127.7, 127.0, 126.6, 126.3, 126.2, 125.6, 123.3, 123.1, 110.0, 109.4, 43.5, 42.8, 33.7, 26.3. MS (ESI): [M + H]^+^ 411.2.

##### 6-(2,3-Dihydrobenzo[b][1,4]dioxin-6-yl)-N-(piperidin-4-ylmethyl)-benzofuran-2-carboxamide hydrochloride (**69**)

^1^H NMR (400 MHz, DMSO-*d*_6_) *δ* 8.92–8.80 (m 2H), 8.60–8.50 (m, 1H), 7.82–7.76 (m, 2H), 7.60–7.55 (m, 2H), 7.35 (s, 1H), 7.25–7.16 (m, 3H), 7.09 (s, 1H), 6.95 (d, *J* = 8.3 Hz, 1H), 4.28 (s, 4H), 3.28–3.13 (m, 4H), 2.88–2.75 (m, 2H), 1.91–1.65 (m, 3H), 1.46–1.28 (m, 2H). ^13^C NMR (100 MHz, DMSO-*d*_6_) *δ* 158.7, 144.1, 143.8, 139.3, 139.2, 133.5, 126.4, 123.3, 123.1, 120.4, 118.0, 117.5, 115.9, 109.8, 109.4, 64.6, 64.5, 43.9, 43.2, 34.1, 26.7. MS (ESI): [M + H]^+^ 393.2.

##### 6-(4-(Methylsulfonyl)phenyl)-N-(piperidin-4-ylmethyl)-benzofuran-2-carboxamide hydrochloride (**70**)

^1^H NMR (400 MHz, DMSO-*d*_6_) *δ* 8.92 (t, *J* = 6.1 Hz, 1H), 8.81–8.73 (m, 1H), 8.50–8.39 (m, 1H), 8.06–7.98 (m, 5H), 7.90 (d, *J* = 8.2 Hz, 1H), 7.73 (d, *J* = 8.3 Hz, 1H), 7.62 (s, 1H), 3.29–3.19 (m, 7H), 2.89–2.75 (m, 2H), 1.93–1.74 (m, 3H), 1.45–1.27 (m, 2H). ^13^C NMR (100 MHz, DMSO-*d*_6_) *δ* 158.2, 154.8, 150.2, 144.8, 139.8, 137.3, 128.0, 127.7, 127.5, 123.4, 123.3, 110.4, 109.3, 43.6, 43.5, 42.8, 33.6, 26.3. MS (ESI): [M + H]^+^ 413.1.

##### 6-(4-(Benzyloxycarbonylaminomethyl)phenyl)-N-(piperidin-4-ylmethyl)-benzofuran-2-carboxamide hydrochloride (**71**)

^1^H NMR (400 MHz, DMSO-*d*_6_) *δ* 9.06–8.95 (m, 1H), 8.94–8.90 (m, 1H), 8.78–8.62 (m, 1H), 7.95–7.79 (m, 3H), 7.69 (d, *J* = 8.0 Hz, 2H), 7.65–7.57 (m, 2H), 7.43–7.25 (m, 7H), 5.05 (s, 2H), 4.25 (d, *J* = 6.0 Hz, 2H), 3.72–3.62 (m, 1H), 3.50–3.40 (m, 1H), 3.28–3.18 (m, 4H), 2.88–2.72 (m, 2H), 1.88–1.72 (m, 3H), 1.45–1.30 (m, 2H). ^13^C NMR (100 MHz, DMSO-*d*_6_) *δ* 158.2, 156.4, 155.0, 149.6, 139.3, 139.1, 138.4, 137.1, 128.4, 127.8, 127.7, 127.7, 127.0, 126.4, 123.0, 122.9, 109.4, 109.3, 65.4, 43.5, 43.5, 42.7, 33.6, 26.2. MS (ESI): [M + H]^+^ 498.2.

##### 6-(4-(Aminomethyl)phenyl)-N-(piperidin-4-ylmethyl)-benzofuran-2-carboxamide hydrochloride (**72**)

^1^H NMR (400 MHz, DMSO-*d*_6_) *δ* 9.05–8.95 (m, 1H), 8.92 (t, *J* = 5.9 Hz, 1H), 8.75–8.65 (m, 1H), 8.50 (br, 3H), 7.91 (s, 1H), 7.88–7.75 (m, 3H), 7.69–7.65 (m, 1H), 7.64–7.58 (m, 3H), 4.11–4.02 (m, 2H), 3.29–3.15 (m, 4H), 2.89–2.73 (m, 2H), 1.89–1.70 (m, 3H), 1.43–1.30 (m, 2H). ^13^C NMR (100 MHz, DMSO-*d*_6_) *δ* 158.3, 155.0, 149.8, 139.9, 138.6, 133.6, 129.7, 127.2, 126.7, 123.2, 123.0, 109.7, 109.3, 43.5, 42.8, 41.9, 33.7, 26.6. MS (ESI): [M + H]^+^ 364.2.

##### 6-(4-Cyanophenyl)-N-(piperidin-4-ylmethyl)-benzofuran-2-carboxamide hydrochloride (**73**)

^1^H NMR (400 MHz, DMSO-*d*_6_) *δ* 8.92 (t, *J* = 5.7 Hz, 1H), 8.81–8.70 (m, 1H), 8.52–8.37 (m, 1H), 8.01 (s, 1H), 7.99–7.92 (m, 3H), 7.89 (d, *J* = 8.2 Hz, 1H), 7.73 (d, *J* = 8.3 Hz, 1H), 7.61 (s, 1H), 3.28–3.10 (m, 4H), 2.88–2.75 (m, 2H), 1.89–1.75 (m, 3H), 1.42–1.30 (m, 2H). ^13^C NMR (100 MHz, DMSO-*d*_6_) *δ* 158.2, 154.9, 150.2, 144.3, 137.2, 133.0, 128.0, 127.6, 123.4, 123.2, 118.9, 110.3, 110.2, 109.3, 43.5, 42.9, 33.6, 26.3. MS (ESI): [M + H]^+^ 360.2.

#### 3.1.4. Synthetic Methods for Compounds **29**, **30**, **33**, **34**, **51**–**52**, and **63**–**66**

An amidation and a Suzuki coupling reaction, as described in the General synthetic procedure-A, were used for synthesis of benzothiophene- or benzofuran-2-carboxamide **85**. Compounds **29**, **30**, **33**, **34**, **51**–**52**, and **63**–**66** were prepared using an additional amidation reaction followed by BOC deprotection, following the General synthetic procedure-A, as a hydrochloric salt.

##### 6-(4-(Piperidine-1-carbonyl)phenyl)-N-(piperidin-4-ylmethyl)-benzo[b]thiophene-2-carboxamide hydrochloride (**29**)

^1^H NMR (400 MHz, DMSO-*d*_6_) *δ* 9.05–8.90 (m, 2H), 8.71–8.59 (m, 1H), 8.38 (s, 1H), 8.16 (s, 1H), 8.02 (d, *J* = 8.3 Hz, 1H), 7.83 (d, *J* = 6.7 Hz, 2H), 7.78 (d, *J* = 8.4 Hz, 1H), 7.47 (d, *J* = 6.7 Hz, 2H), 3.65–3.58 (m, 2H), 3.36–3.18 (m, 6H), 2.85–2.75 (m, 2H), 1.89–1.75 (m, 3H), 1.63–1.36 (m, 8H). ^13^C NMR (100 MHz, DMSO-*d*_6_) *δ* 168.6, 161.6, 141.1, 140.6, 140.4, 138.7, 137.3, 135.6, 127.4, 127.0, 125.6, 124.5, 124.1, 120.8, 48.8, 44.0, 42.8, 33.7, 26.3, 24.7, 24.1. MS (ESI): [M + H]^+^ 462.2.

##### 6-(4-(Pyrroline-1-carbonyl)phenyl)-N-(piperidin-4-ylmethyl)-benzo[b]thiophene-2-carboxamide hydrochloride (**30**)

^1^H NMR (400 MHz, DMSO-*d*_6_) *δ* 8.97 (t, *J* = 5.8 Hz, 1H), 8.94–8.84 (m, 1H), 8.65–8.55 (m, 1H), 8.39 (s, 1H), 8.16 (s, 1H), 8.02 (d, *J* = 8.4 Hz, 1H), 7.88–7.75 (m, 3H), 7.63 (d, *J* = 8.1 Hz, 2H), 3.50–3.41 (m, 4H), 3.28–3.18 (m, 4H), 2.85–2.78 (m, 2H), 1.93–1.77 (m, 7H), 1.45–1.30 (m, 2H). ^13^C NMR (100 MHz, DMSO-*d*_6_) *δ* 168.3, 162.0, 141.5, 141.2, 141.1, 139.1, 137.7, 136.7, 128.3, 127.2, 126.0, 124.9, 124.5, 121.3, 49.4, 46.4, 44.5, 43.3, 34.1, 26.7, 25.0, 24.4. MS (ESI): [M + H]^+^ 448.2.

##### 6-(4-(4-(Piperidin-1-yl)piperidin-1-carbonyl)phenyl)-N-(piperidin-4-ylmethyl)-benzo[b]thiophene-2-carboxamide hydrochloride (**33**)

^1^H NMR (400 MHz, DMSO-*d*_6_) *δ* 10.68–10.55 (m, 1H), 9.05–8.95 (m, 2H), 8.78–8.65 (m, 1H), 8.39 (s, 1H), 8.18 (s, 1H), 8.02 (d, *J* = 8.3 Hz, 1H), 7.84 (d, *J* = 7.8 Hz, 2H), 7.78 (d, *J* = 8.3 Hz, 1H), 7.52 (d, *J* = 7.8 Hz, 2H), 3.69–3.33 (m, 6H), 3.30–3.16 (m, 4H), 2.96–2.75 (m, 5H), 2.24–2.03 (m, 2H), 1.93–1.66 (m, 10H), 1.45–1.32 (m, 3H). ^13^C NMR (100 MHz, DMSO-*d*_6_) *δ* 168.6, 161.6, 141.1, 140.8, 140.7, 138.7, 137.2, 134.9, 127.6, 127.0, 125.6, 124.5, 124.1, 120.9, 70.5, 62.3, 60.2, 48.9, 44.0, 42.8, 33.7, 26.3, 22.5, 21.6. MS (ESI): [M + H]^+^ 545.3.

##### 6-(4-(4-(Pyrrolidin-1-yl)piperidin-1-carbonyl)phenyl)-N-(piperidin-4-ylmethyl)-benzo[b]thiophene-2-carboxamide hydrochloride (**34**)

^1^H NMR (400 MHz, DMSO-*d*_6_) *δ* 11.21 (br, 1H), 9.05–8.95 (m, 2H), 8.76–8.56 (m, 1H), 8.39 (s, 1H), 8.17 (s, 1H), 8.02 (d, *J* = 8.3 Hz, 1H), 7.85 (d, *J* = 7.9 Hz, 2H), 7.78 (d, *J* = 8.3 Hz, 1H), 7.50 (d, *J* = 7.8 Hz, 2H), 3.75–3.65 (m, 2H), 3.52–3.32 (m, 4H), 3.28–3.16 (m, 4H), 3.09–2.98 (m, 2H), 2.88–2.75 (m, 3H), 2.15–1.66 (m, 11H), 1.48–1.34 (m, 2H). ^13^C NMR (100 MHz, DMSO-*d*_6_) *δ* 168.7, 161.6, 141.1, 140.8, 140.7, 138.7, 137.2, 134.9, 127.6, 127.1, 125.6, 124.5, 124.1, 120.9, 60.5, 50.3, 44.0, 42.8, 33.7, 26.3, 22.7. MS (ESI): [M + H]^+^ 531.3.

##### 5-(4-(Piperidine-1-carbonyl)phenyl)-N-(piperidin-4-ylmethyl)-benzo[b]thiophene-2-carboxamide hydrochloride (**51**)

^1^H NMR (400 MHz, DMSO-*d*_6_) *δ* 8.99 (t, *J* = 5.8 Hz, 1H), 8.85–8.75 (m, 1H), 8.52–8.40 (m, 1H), 8.24 (s, 1H), 8.16 (s, 1H), 8.11 (d, *J* = 8.4 Hz, 1H), 7.85–7.75 (m, 3H), 7.48 (d, *J* = 8.2 Hz, 2H), 3.66–3.47 (m, 2H), 3.31–3.13 (m, 6H), 2.88–2.75 (m, 2H), 1.90–1.76 (m, 3H), 1.68–1.34 (m, 8H). ^13^C NMR (100 MHz, DMSO-*d*_6_) *δ* 168.6, 161.6, 140.9, 140.7, 139.9, 139.6, 136.5, 135.5, 127.4, 127.0, 125.2, 124.9, 123.4, 123.0, 44.0, 42.9, 33.7, 26.3, 24.1. MS (ESI): [M + H]^+^ 462.2.

##### 5-(4-(Pyrroline-1-carbonyl)phenyl)-N-(piperidin-4-ylmethyl)-benzo[b]thiophene-2-carboxamide hydrochloride (**52**)

^1^H NMR (400 MHz, DMSO-*d*_6_) *δ* 9.05–8.95 (m, 1H), 8.87–8.75 (m, 1H), 8.56–8.45 (m, 1H), 8.24 (s, 1H), 8.17 (s, 1H), 8.11 (d, *J* = 8.5 Hz, 1H), 7.85–7.75 (m, 3H), 7.63 (d, *J* = 8.1 Hz, 2H), 3.54–3.42 (m, 4H), 3.28–3.16 (m, 4H), 2.90–2.78 (m, 2H), 1.91–1.77 (m, 7H), 1.49–1.30 (m, 2H). ^13^C NMR (100 MHz, DMSO-*d*_6_) *δ* 167.9, 161.6, 141.0, 140.9, 139.9, 139.7, 136.4, 136.1, 127.8, 126.7, 125.2, 124.9, 123.4, 123.0, 48.9, 46.0, 44.0, 42.8, 33.7, 26.3, 26.0, 23.9. MS (ESI): [M + H]^+^ 448.2.

##### 6-(4-((Pyrrolidine-1-yl)carbonyl)phenyl)-N-(piperidin-4-ylmethyl)-benzofuran-2-carboxamide hydrochloride (**63**)

^1^H NMR (400 MHz, DMSO-*d*_6_) *δ* 9.18–9.10 (m, 1H), 8.95 (t, *J* = 5.6 Hz, 1H), 8.89–8.80 (m, 1H), 7.94 (s, 1H), 7.85 (d, *J* = 8.2 Hz, 1H), 7.80 (d, *J* = 7.6 Hz, 2H), 7.68 (d, *J* = 8.3 Hz, 1H), 7.66–7.55 (m, 3H), 3.51–3.34 (m, 4H), 3.28–3.15 (m, 4H), 2.84–2.74 (m, 2H), 1.94–1.70 (m, 7H), 1.46–1.32 (m, 2H). ^13^C NMR (100 MHz, DMSO-*d*_6_) *δ* 167.9, 158.2, 154.9, 149.8, 141.0, 138.4, 136.3, 127.9, 126.9, 123.2, 123.0, 109.8, 109.3, 46.0, 43.5, 42.7, 33.7, 26.2, 26.0, 23.9. MS (ESI): [M + H]^+^ 432.2.

##### 6-(4-((Piperidine-1-yl)carbonyl)phenyl)-N-(piperidin-4-ylmethyl)-benzofuran-2-carboxamide hydrochloride (**64**)

^1^H NMR (400 MHz, DMSO-*d*_6_) *δ* 8.98–8.88 (m, 2H), 8.68–8.55 (m, 1H), 7.93 (s, 1H), 7.86–7.78 (m, 3H), 7.74–7.57 (m, 2H), 7.47 (d, *J* = 7.9 Hz, 2H), 3.35–3.14 (m, 8H), 2.84–2.74 (m, 2H), 1.89–1.75 (m, 3H), 1.65–1.53 (m, 6H), 1.45–1.32 (m, 2H). ^13^C NMR (100 MHz, DMSO-*d*_6_) *δ* 168.6, 158.2, 154.9, 149.8, 140.6, 138.4, 135.7, 127.5, 127.1, 126.8, 123.2, 123.0, 109.7, 109.3, 43.5, 42.8, 33.6, 26.2, 24.7, 24.1, 23.2. MS (ESI): [M + H]^+^ 446.2.

##### 6-(4-((Azepane-1-yl)carbonyl)phenyl)-N-(piperidin-4-ylmethyl)-benzofuran-2-carboxamide hydrochloride (**65**)

^1^H NMR (400 MHz, DMSO-*d*_6_) *δ* 8.98–8.90 (m, 2H), 8.68–8.55 (m, 1H), 7.93 (s, 1H), 7.85 (d, *J* = 8.2 Hz, 1H), 7.80 (d, *J* = 8.0 Hz, 2H), 7.71–7.53 (m, 2H), 7.46 (d, *J* = 8.0 Hz, 2H), 3.36–3.17 (m, 8H), 2.85–2.75 (m, 2H), 1.89–1.70 (m, 5H), 1.66–1.50 (m, 6H), 1.42–1.30 (m, 2H). ^13^C NMR (100 MHz, DMSO-*d*_6_) *δ* 169.9, 158.2, 154.9, 149.8, 140.2, 138.4, 136.6, 127.1, 127.0, 126.8, 123.1, 123.0, 109.7, 109.3, 51.0, 45.4, 43.5, 42.8, 33.6, 27.2, 26.8, 26.2, 26.0, 25.8. MS (ESI): [M + H]^+^ 460.3.

##### 6-(4-((1,4′-Bipiperidine-1-yl)carbonyl)phenyl)-N-(piperidin-4-ylmethyl)-benzofuran-2-carboxamide hydrochloride (**66**)

^1^H NMR (400 MHz, DMSO-*d*_6_) *δ* 10.49–10.40 (m, 1H), 8.98–8.85 (m, 2H), 8.64–8.50 (m, 1H), 7.95 (s, 1H), 7.88–7.82 (m, 3H), 7.69 (d, *J* = 8.3 Hz, 1H), 7.61 (s, 1H), 7.53 (d, *J* = 7.9 Hz, 2H), 3.40–3.19 (m, 9H), 2.99–2.71 (m, 6H), 2.20–2.20 (m, 2H), 1.87–1.65 (m, 10H), 1.45–1.30 (m, 3H). ^13^C NMR (100 MHz, DMSO-*d*_6_) *δ* 168.6, 158.2, 154.9, 149.8, 141.0, 138.3, 134.9, 127.6, 127.1, 126.9, 123.2, 123.0, 109.8, 109.3, 70.5, 62.3, 60.2, 49.0, 43.5, 42.8, 33.6, 26.2, 22.5, 21.6. MS (ESI): [M + H]^+^ 529.3.

Compounds **31**, **32**, **35**, **36**, **55**, **56** and **74**–**77** were synthesized using the general synthetic procedure-A, starting from benzothiophene- or benzofuran-2-carboxamide **84**, as a hydrochloric salt.

##### 6-((4-(Piperidin-1-yl)piperidin-1-yl)phenyl)-N-(piperidin-4-ylmethyl)-benzo[b]thiophene-2-carboxamide hydrochloride (**31**)

^1^H NMR (400 MHz, DMSO-*d*_6_) *δ* 10.37 (br, 1H), 8.96–8.85 (m, 1H), 8.76 (t, *J* = 6.0 Hz, 1H), 8.66–8.49 (m, 1H), 7.97 (s, 1H), 7.75 (d, *J* = 8.9 Hz, 1H), 7.53 (s, 1H), 7.23 (d, *J* = 8.7 Hz, 1H), 3.99–3.92 (m, 2H), 3.45–3.36 (m, 2H), 3.28–3.20 (m, 2H), 3.15 (t, *J* = 5.6 Hz, 2H), 2.95–2.80 (m, 6H), 2.24–2.15 (m, 2H), 1.89–1.75 (m, 10H), 1.73–1.65 (m, 1H), 1.45–1.32 (m, 3H). ^13^C NMR (100 MHz, DMSO-*d*_6_) *δ* 161.9, 148.3, 142.3, 139.0, 135.2, 125.5, 124.5, 119.4, 116.2, 62.2, 50.6, 48.9, 43.9, 42.7, 33.7, 26.3, 25.2, 22.5, 21.6. MS (ESI): [M + H]^+^ 441.3.

##### 6-((4-(Pyrrolidin-1-yl)piperidin-1-yl)phenyl)-N-(piperidin-4-ylmethyl)-benzo[b]thiophene-2-carboxamide hydrochloride (**32**)

^1^H NMR (400 MHz, DMSO-*d*_6_) *δ* 10.87 (br, 1H), 8.88–8.80 (m, 1H), 8.75 (t, *J* = 5.8 Hz, 1H), 8.60–8.50 (m, 1H), 7.96 (s, 1H), 7.74 (d, *J* = 9.0 Hz, 1H), 7.53 (s, 1H), 7.23 (d, *J* = 8.6 Hz, 1H), 3.99–3.90 (m, 2H), 3.54–3.44 (m, 2H), 3.33–3.20 (m, 3H), 3.15 (t, *J* = 5.8 Hz, 2H), 3.10–3.00 (m, 2H), 2.89–2.75 (m, 4H), 2.18–2.10 (m, 2H), 1.99–1.76 (m, 9H), 1.50–1.25 (m, 2H). MS (ESI): [M + H]^+^ 427.2.

##### 6-((4-(Piperidin-1-yl)phenyl)amino)-N-(piperidin-4-ylmethyl)-benzo[b]thiophene-2-carboxamide hydrochloride (**35**)

^1^H NMR (400 MHz, DMSO-*d*_6_) *δ* 12.58 (br, 1H), 9.13–8.87 (m, 2H), 8.84–8.57 (m, 2H), 8.02 (s, 1H), 7.85–7.72 (m, 3H), 7.67 (s, 1H), 7.25 (d, *J* = 8.9 Hz, 2H), 7.18 (dd, *J* = 8.7, 1.9 Hz, 1H), 3.52–3.32 (m, 4H), 3.26–3.20 (m, 4H), 2.88–2.74 (m, 2H), 2.23–2.05 (m, 2H), 1.92–1.53 (m, 7H), 1.45–1.28 (m, 2H). ^13^C NMR (100 MHz, DMSO-*d*_6_) *δ* 161.9, 143.9, 142.1, 141.3, 136.7, 135.0, 133.1, 125.8, 124.6, 122.6, 117.3, 117.0, 109.6, 108.4, 56.1, 43.9, 42.7, 33.7, 26.3, 22.9, 20.8. MS (ESI): [M + H]^+^ 449.2.

##### 6-(4-(4-Chlorophenoxyl)phenoxyl)-N-(piperidin-4-ylmethyl)-benzo[b]thiophene-2-carboxamide hydrochloride (**36**)

^1^H NMR (400 MHz, DMSO-*d*_6_) *δ* 8.92–8.80 (m, 2H), 8.61–8.45 (m, 1H), 8.08 (s, 1H), 7.93 (d, *J* = 8.7 Hz, 1H), 7.61 (s, 1H), 7.43 (d, *J* = 8.8 Hz, 2H), 7.17–7.01 (m, 7H), 3.27–3.14 (m, 4H), 2.88–2.75 (m, 2H), 1.89–1.75 (m, 3H), 1.39–1.30 (m, 2H). ^13^C NMR (100 MHz, DMSO-*d*_6_) *δ* 161.6, 156.2, 156.0, 152.3, 152.0, 141.8, 139.1, 135.1, 129.9, 127.0, 126.5, 124.3, 120.8, 119.7, 117.2, 111.0, 44.0, 42.8, 33.7, 26.3. MS (ESI): [M + H]^+^ 493.1.

##### 5-(4-(Tert-butyl)phenyl)-N-(piperidin-4-ylmethyl)-benzo[b]thiophene-2-carboxamide hydrochloride (**55**)

^1^H NMR (400 MHz, DMSO-*d*_6_) *δ* 9.03–8.92 (m, 1H), 8.83 (t, *J* = 5.8 Hz, 1H), 8.69–8.60 (m, 1H), 7.96 (s, 1H), 7.79 (d, *J* = 8.8 Hz, 1H), 7.49 (d, *J* = 2.0 Hz, 1H), 7.26 (d, *J* = 8.6 Hz, 2H), 7.17 (dd, *J* = 8.8, 2.1 Hz, 1H), 7.05 (d, *J* = 8.6 Hz, 2H), 3.31–3.10 (m, 4H), 2.88–2.75 (m, 2H), 1.89–1.75 (m, 3H), 1.45–1.35 (m, 2H), 1.25 (s, 9H). ^13^C NMR (100 MHz, DMSO-*d*_6_) *δ* 161.8, 142.3, 141.7, 140.8, 140.3, 140.3, 131.4, 125.8, 124.5, 123.3, 118.4, 116.9, 109.6, 44.0, 42.8, 33.8, 33.7, 31.3, 26.3. MS (ESI): [M + H]^+^ 422.2.

##### 5-((4-(Trifluoromethoxyl)phenyl)amino)-N-(piperidin-4-ylmethyl)-benzo[b]thiophene-2-carboxamide hydrochloride (**56**)

^1^H NMR (400 MHz, DMSO-*d*_6_) *δ* 8.86–8.75 (m, 2H), 8.58–8.43 (m, 2H), 7.98 (s, 1H), 7.86 (d, *J* = 8.7 Hz, 1H), 7.59 (s, 1H), 7.25–7.10 (m, 5H), 3.27–3.13 (m, 4H), 2.88–2.78 (m, 2H), 1.88–1.76 (m, 3H), 1.45–1.30 (m, 2H). MS (ESI): [M + H]^+^ 450.1.

##### 6-((4-(Piperidin-1-yl)phenyl)amino)-N-(piperidin-4-ylmethyl)-benzofuran-2-carboxamide hydrochloride (**74**)

^1^H NMR (400 MHz, DMSO-*d*_6_) *δ* 12.39 (br, 1H), 9.01–8.83 (m, 2H), 8.72–8.48 (m, 2H), 7.79–7.68 (m, 2H), 7.59 (d, *J* = 8.5 Hz, 1H), 7.43 (s, 1H), 7.33–7.16 (m, 3H), 7.08 (d, *J* = 8.5 Hz, 1H), 3.40–3.06 (m, 8H), 2.86–2.72 (m, 2H), 2.19–2.05 (m, 2H), 1.87–1.56 (m, 7H), 1.39–1.25 (m, 2H). ^13^C NMR (100 MHz, DMSO-*d*_6_) *δ* 158.5, 156.1, 147.3, 146.9, 145.7, 133.9, 122.9, 120.9, 118.1, 117.3, 113.5, 109.9, 94.7, 50.5, 45.4, 42.8, 33.7, 26.2, 25.5, 23.9. MS (ESI): [M + H]^+^ 433.3.

6-((4-Trifuluoromethoxyl)phenyl)amino)-N-(piperidin-4-ylmethyl)-benzofuran-2-carboxamide hydrochloride (**75**)

^1^H NMR (400 MHz, DMSO-*d*_6_) *δ* 8.86–8.78 (m, 1H), 8.76 (s, 1H), 8.67 (t, *J* = 5.8 Hz, 1H), 8.57–8.43 (m, 1H), 7.59 (d, *J* = 8.6 Hz, 1H), 7.42 (s, 1H), 7.28–7.15 (m, 4H), 7.06 (d, *J* = 8.5 Hz, 1H), 3.27–3.10 (m, 4H), 2.86–2.75 (m, 2H), 1.88–1.73 (m, 3H), 1.41–1.26 (m, 2H). ^13^C NMR (100 MHz, DMSO-*d*_6_) *δ* 158.4, 155.6, 147.8, 142.8, 142.3, 123.2, 122.3, 120.1, 117.9, 115.3, 109.7, 98.0, 43.3, 42.8, 33.7, 26.2. MS (ESI): [M + H]^+^ 434.2.

##### 6-(4-Methoxylphenoxyl)-N-(piperidin-4-ylmethyl)-benzofuran-2-carboxamide hydrochloride (**76**)

^1^H NMR (400 MHz, DMSO-*d*_6_) *δ* 9.08–8.95 (m, 1H), 8.81–8.63 (m, 2H), 7.70 (d, *J* = 8.5 Hz, 1H), 7.52 (s, 1H), 7.10 (s, 1H), 7.08–6.92 (m, 5H), 3.75 (s, 3H), 3.27–3.07 (m, 4H), 2.88–2.72 (m, 2H), 1.88–1.72 (m, 3H), 1.46–1.28 (m, 2H). ^13^C NMR (100 MHz, DMSO-*d*_6_) *δ* 158.1, 157.7, 155.8, 154.9, 149.3, 149.2, 123.5, 122.2, 120.8, 115.2, 114.7, 109.4, 100.4, 55.5, 43.3, 42.7, 33.6, 26.2. MS (ESI): [M + H]^+^ 381.2.

##### 6-(4-(4-Cholorophenoxyl)phenoxyl)-N-(piperidin-4-ylmethyl)-benzofuran-2-carboxamide hydrochloride (**77**)

^1^H NMR (400 MHz, DMSO-*d*_6_) *δ* 8.94–8.77 (m, 2H), 8.62–8.42 (m, 1H), 7.75 (d, *J* = 8.5 Hz, 1H), 7.53 (s, 1H), 7.42 (d, *J* = 8.5 Hz, 2H), 7.25 (s, 1H), 7.15–6.98 (m, 7H), 3.28–3.15 (m, 4H), 2.88–2.75 (m, 2H), 1.89–1.72 (m, 3H), 1.40–1.30 (m, 2H). ^13^C NMR (100 MHz, DMSO-*d*_6_) *δ* 158.1, 156.6, 156.2, 154.8, 152.5, 151.9, 149.4, 129.8, 126.9, 123.7, 122.8, 120.8, 120.6, 119.7, 115.5, 109.4, 101.5, 43.3, 42.8, 33.6, 26.2. MS (ESI): [M + H]^+^ 477.2.

##### 6-(4-(Piperidin-1-yl)phenyl)-N-(piperidin-4-ylmethyl)-benzofuran-3-carboxamide hydrochloride (**57**)

It was synthesized using the general synthetic procedure-A, starting from 6-bromo-benzofuran-3-carboxylic acid **86**, as a hydrochloric salt. ^1^H NMR (400 MHz, DMSO-*d*_6_) *δ* 9.01–8.89 (m, 1H), 8.75–8.60 (m, 4H), 8.13 (d, *J* = 8.2 Hz, 1H), 8.03–7.89 (m, 4H), 7.71 (d, *J* = 8.3 Hz, 1H), 3.55–3.50 (m, 2H), 3.34–3.07 (m, 6H), 2.85–2.75 (m, 2H), 2.10–1.58 (m, 8H), 1.47–1.28 (m, 3H). ^13^C NMR (100 MHz, DMSO-*d*_6_) *δ* 162.0, 155.4, 148.3, 148.1, 135.9, 128.3, 127.0, 123.4, 122.9, 122.3, 116.9, 114.9, 109.8, 66.4, 43.4, 42.8, 33.8, 26.3, 25.0, 23.1. MS (ESI): [M + H]^+^ 418.2.

### 3.2. Plasmids and Peptides

The cDNA for the human AF9 AHD domain (475–568) and ENL AHD domain (489–559) was synthesized (by Genscript, Piscataway, NJ, USA) and inserted into the pMAL c5X expression plasmid. The DOT1L peptide (Biotin-AHX-NKLPVSIPLASVVLPSRAERARST) and AF4 peptide (Biotin-AHX-QSLMVKITLDLLSRIPQPPGK) for the Alpha assay were purchased from Genscript.

### 3.3. Protein Expression and Purification

The AF9 or ENL expression plasmid was used to transform *E. coli* BL21(DE3) strain (Novagen, Madison, WI, USA), and protein expression was induced by adding 0.4 mM isopropyl β-D-1-thiogalactopyranoside (IPTG) at 16 °C overnight. The cells were collected and lysed using a French press (GlenMills, Clifton, NJ, USA) in a lysis buffer (50 mM HEPES, 200 mM NaCl, 1 mM DTT, pH 7.4). Upon centrifugation, the supernatant was applied to an amylose resin column (GE Healthcare) and the recombinant protein with a N-terminal maltose-binding protein (MBP) tag was eluted with 20 mM HEPES, 10 mM Maltose, pH 7.4, which was further purified to be > 95% (SDS-PAGE) using a size exclusion column (HiLoad 16/60 Superdex 200, GE Healthcare, Chicago, IL, USA).

### 3.4. AlphaLisa Assays

Alpha assays were developed in our previous publication [26], using a Perkin-Elmer AlphaLisa anti-MBP kit, which contains streptavidin donor beads and anti-MBP acceptor beads. Briefly, the assay was performed using a MBP-protein (5 nM), a biotinylated peptide (40 nM), and increasing concentrations of a compound in 25 µL buffer (PBS with 0.5% BSA, pH 7.5) in 384-well plates according to the manufacturer’s protocol and measured with a Tecan SPARK microplate reader. Data were imported into Prism (version 5.0), and IC_50_ values from 3 independent experiments with standard deviation were obtained by using a standard dose-response curve fitting.

### 3.5. Pull-Down Assay

The pull-down assays were conducted following our previous method [26]. Briefly, the biotinylated DOT1L peptide (0.25 mg/mL) was incubated with streptavidin agarose beads (20 μL) in PBS containing 0.1% Triton X-100 (400 µL) for 3h to yield, after washing, DOT1L-coated beads. MBP-tagged AF9 AHD (0.2 µM, 400 µL) was pre-incubated with a compound for 1 h before adding to the beads. After 6 h incubation at 4 °C, the beads were collected, thoroughly washed, and subjected to SDS-PAGE and analyzed by Western blot using an MBP antibody (#2396, Cell signaling, Danvers, MA, USA).

### 3.6. RNA Extraction and Quantitative Real-Time PCR

RNA extraction and RT-qPCR were performed following our previous method [26]. 10^5^ cells/mL were incubated with a compound for 4 days and the RNA was extracted using an RNeasy mini kit (#74104, Qiagen, Germantown, MD, USA). 100–1000 ng of total RNA was reverse transcribed using iScript™ Reverse Transcription Supermix (Bio-Rad, Hercules, CA, USA), using the manufacturer’s protocol. Quantitative real-time PCR was carried out using Fast SYBR Green Master Mix (Applied Biosystems, Waltham, MA, USA) according to the manufacturer’s instructions. Measurements were performed in triplicate using GAPDH as the reference gene. Real-time PCR was performed using the Biosystems Step One Plus detection system. The following sequences of primers were used:

MYC (forward: 5′-CACCGAGTCGTAGTCGAGGT-3′; reverse: 5′-TTTCGGGTAGTGGAAAACCA-3′);

HoxA9 (forward: 5′-TACGTGGACTCGTTCCTGCT-3′; reverse: 5′-CGTCGCCTTGGACTGGAAG-3′);

Meis1 (forward: 5′-CCAGCATCTAACACACCCTTAC-3′; reverse: 5′-TATGTTGCTGACCGTCCATTAC -3′);

GAPDH (forward: 5′-GCGAGATCCCTCCAAAATCAA-3′; reverse: 5′-GTTCACACCCATGACGAACAT-3′)

### 3.7. Antiproliferation Assay

Proliferation inhibition assays were performed using an XTT assay kit (Biotium, Fremont, CA, USA) following our previous methods [26]. The antiproliferation EC_50_ values were determined using Prism 5 and the reported results were the mean values of at least three independent experiments. The incubation time for all compounds was 7 days.

### 3.8. Statistical Analysis

At least three independent experiments were carried out to generate each dataset. The significance of experimental differences was evaluated using the Student’s *t* test (Prism 5.0, GraphPad Software, Boston, MA, USA). The results are expressed as the mean ± SEM.

## 4. Conclusions

PPIs between AF9/ENL and AF4 or DOT1L are a potential drug target for MLL-r leukemia, as well as other cancers (e.g., AML) driven by SEC-mediated aberrant gene expression. Several indole-carboxamide compounds were identified as novel inhibitors of the AF9-DOT1L interaction. Synthesis and structure–activity relationship studies of 77 compounds show that a 4-piperidin-1-ylphenyl or 4-pyrrolidin-1-ylphenyl R^5^ or R^6^ substituent is essential for these indole- and closely related benzothiophene- and benzofuran-carboxamide compounds to have strong inhibitory activity. The activities of these inhibitors with IC_50_ values as low as 1.6 μM were further confirmed using a pull-down assay. Inhibitors of the AF9-DOT1L interaction also blocked the PPIs between AF9/ENL and AF4 with comparable IC_50_s. Selected inhibitors were found to suppress expression of MLL target genes HoxA9, Meis1 and Myc and inhibit proliferation of MLL-r leukemia and other AML cells with EC_50_s as low as 4.7 μM, while they were inactive against solid tumor Hela cells. This antitumor activity profile is consistent with the roles of AF9/ENL in these tumors, suggesting that their activities are on-target. In conclusion, the identified benzothiophene compounds **24** and **50** are not only useful chemical probes for biological studies of AF9/ENL or SEC, but they also represent novel lead compounds for further drug development against MLL-r leukemia and related cancers.

## Figures and Tables

**Table 1:** Structures and activities of 6-substituted indole compounds against the AF9-DOT1L interaction.

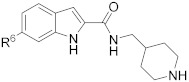 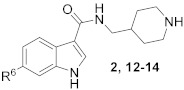					
Cpd #	R^6^	IC_50_ (μM) or % Inhihition at 5 μM	Cpd #	R^6^	IC_50_ (μM) or % Inhihition at 5 μM
**1**	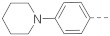	3.3 ± 0.2	**9**	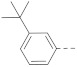	17%
**3**	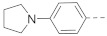	2.8 ± 0.2	**10**	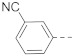	0%
**4**	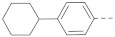	31%	**11**	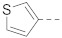	10%
**5**	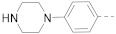	40%	**2**	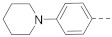	4.5 ± 0.01
**6**	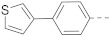	14%	**12**	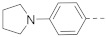	2.9 ± 0.1
**7**	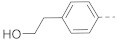	5.6%	**13**	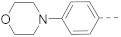	29%
**8**	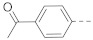	37%	**14**	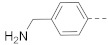	11%

**Table 2:** Structures and activities of 5-substituted indole compounds against the AF9-DOT1L interaction.

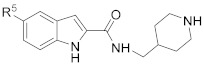 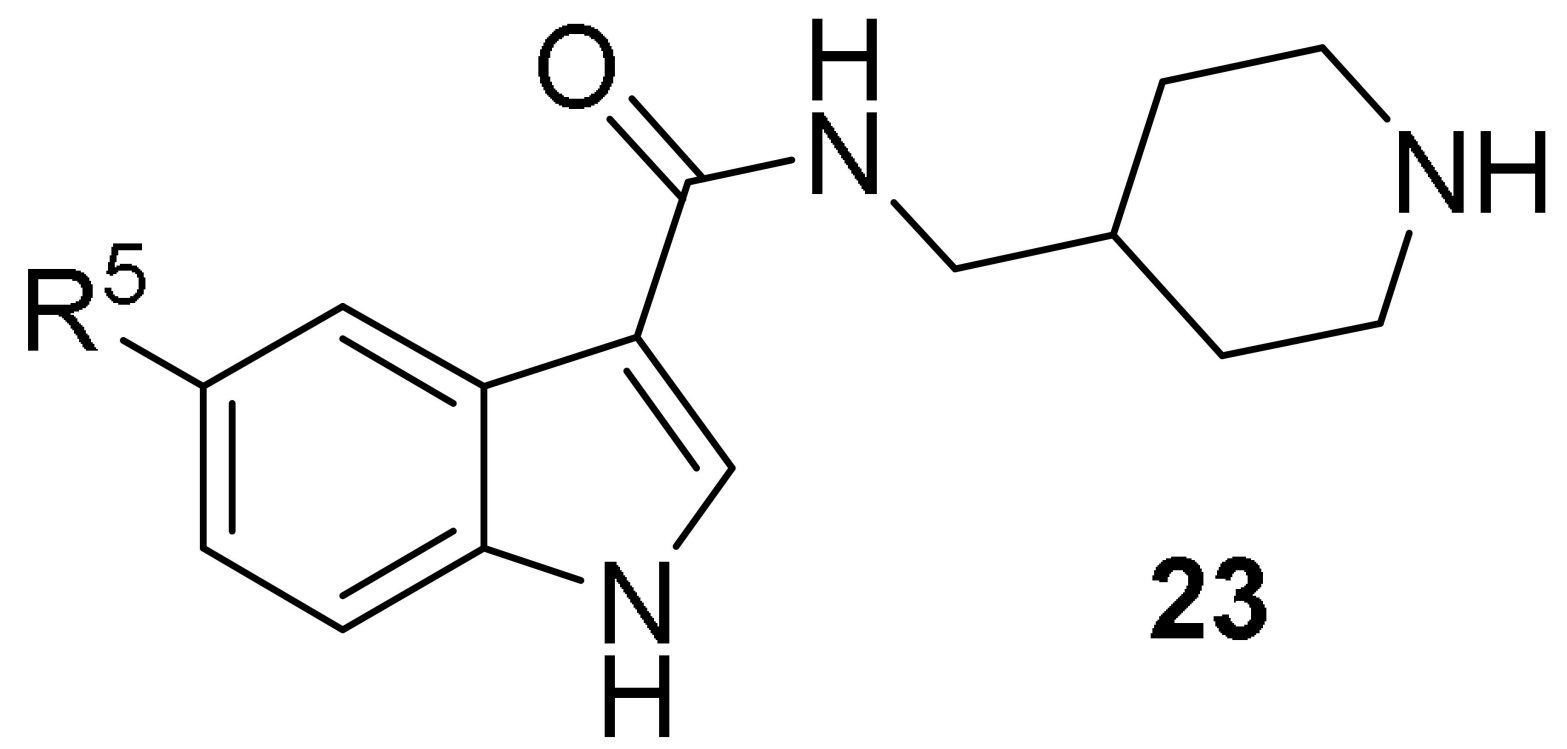					
Cpd #	R^5^	IC_50_ (μM) or % Inhihition at 5 μM	Cpd #	R^5^	IC_50_ (μM) or % Inhihition at 5 μM
**15**	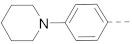	3.6 ± 0.3	**20**	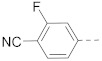	8%
**16**	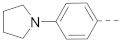	2.7 ± 0.2	**21**	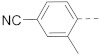	0%
**17**	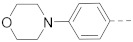	52%	**22**	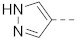	>30
**18**	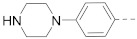	36%	**23**	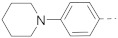	43%
**19**	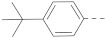	13%			

**Table 3:** Structures and activities of 6-substituted benzothiophene compounds against the AF9-DOT1L interaction.

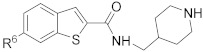					
Cpd #	R^6^	IC_50_ (μM) or % Inhibition at 5 μM	Cpd #	R^6^	IC_50_ (μM) or % Inhihition at 5 μM
**24**	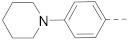	1.6 ± 0.2	**37**	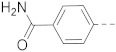	0%
**25**	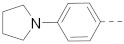	5.3 ± 0.1	**38**	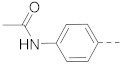	0%
**26**	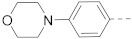	40%	**39**	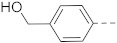	0%
**27**	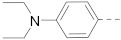	25%	**40**	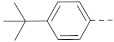	19%
**28**	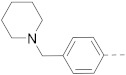	45%	**41**	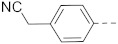	~50%
**29**	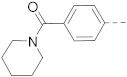	8%	**42**	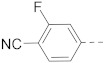	26%
**30**	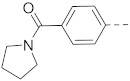	0%	**43**	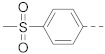	49%
**31**	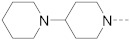	14%	**44**	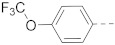	14%
**32**	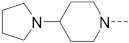	20%	**45**	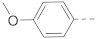	43%
**33**	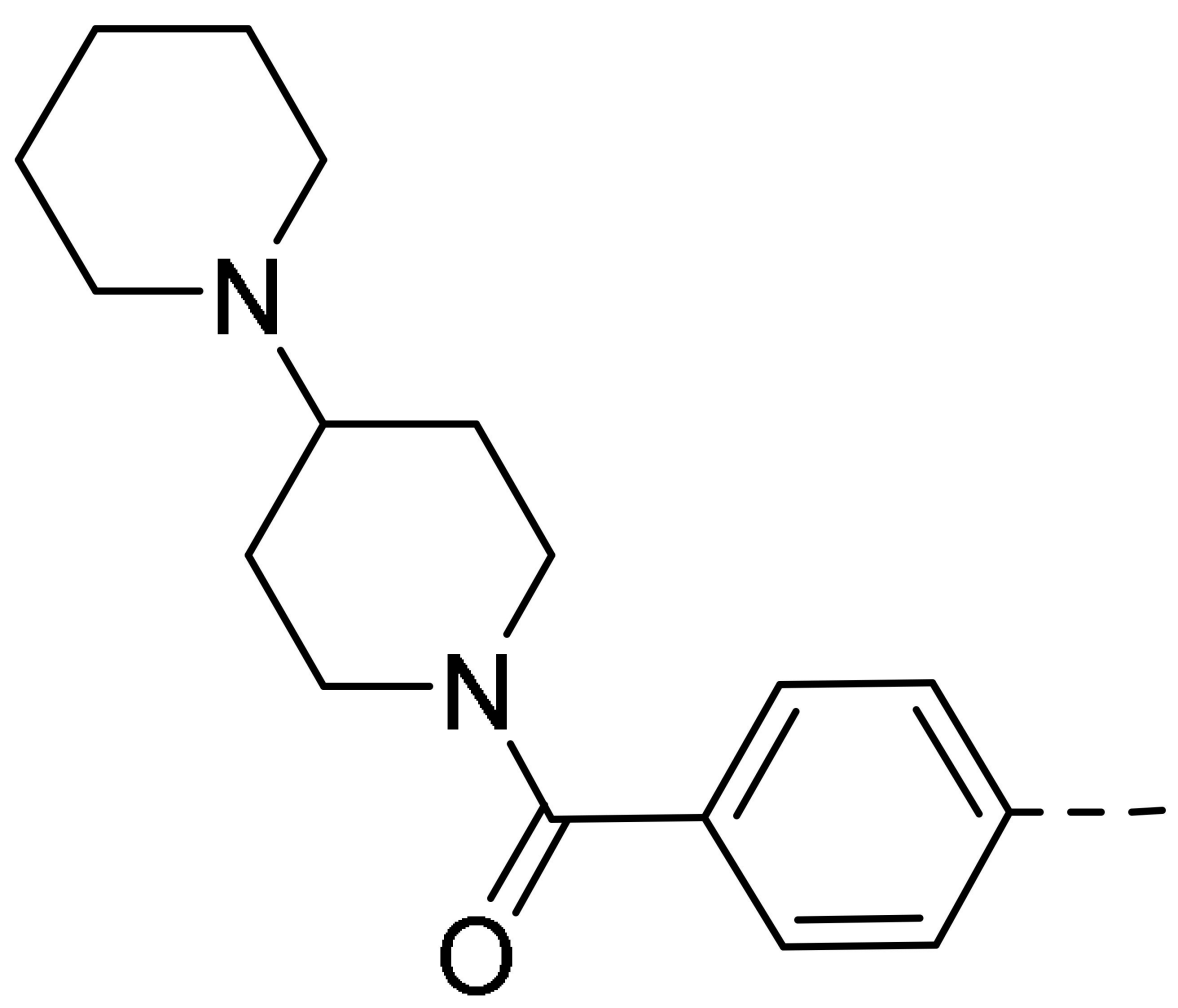	0%	**46**	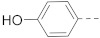	0%
**34**	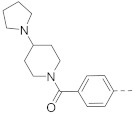	5%	**47**	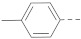	47%
**35**	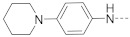	2.3 ± 0.2	**48**	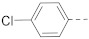	~50%
**36**	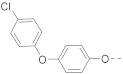	50%	**49**	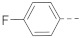	21%

**Table 4:** Structures and activities of 5-substituted benzothiophene compounds against the AF9-DOT1L interaction.

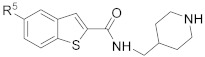		
Cpd #	R^5^	IC_50_ (μM) or % Inhihition at 5 μM
**50**	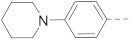	2.5 ± 0.2
**51**	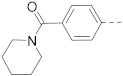	31%
**52**	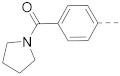	0%
**53**	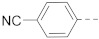	23%
**54**	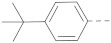	33%
**55**	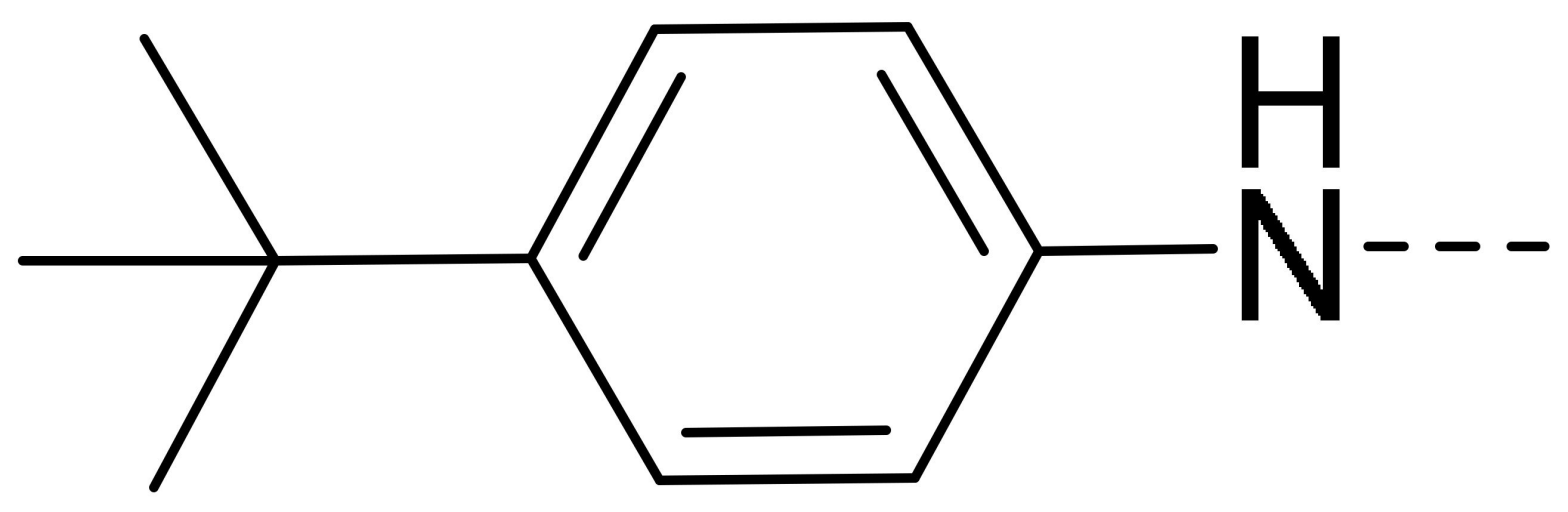	45%
**56**	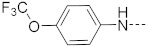	24%

**Table 5:** Structures and activities of 6-substituted benzofuran compounds against the AF9-DOT1L interaction.

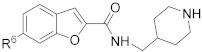 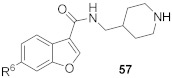					
Cpd #	R^6^	IC_50_ (μM) or % Inhihition at 5 μM	Cpd #	R^6^	IC_50_ (μM) or % Inhihition at 5 μM
**57**	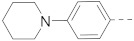	7.2 ± 0.4	**68**	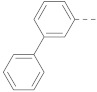	33%
**58**	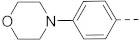	40%	**69**	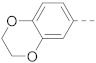	33%
**59**	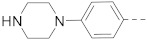	38%	**70**	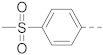	19%
**60**	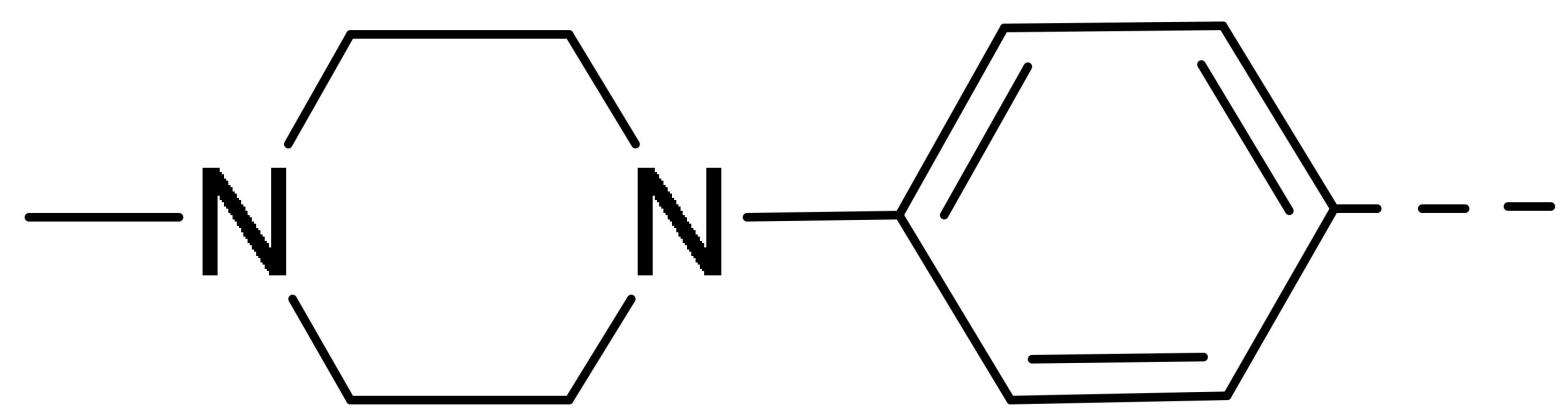	32%	**71**	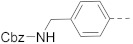	27%
**61**	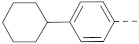	18%	**72**	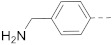	>30
**62**	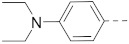	30%	**73**	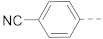	15%
**63**	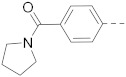	1%	**74**	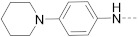	4.6 ± 0.3
**64**	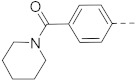	0%	**75**	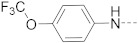	23%
**65**	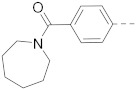	0%	**76**	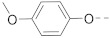	24%
**66**	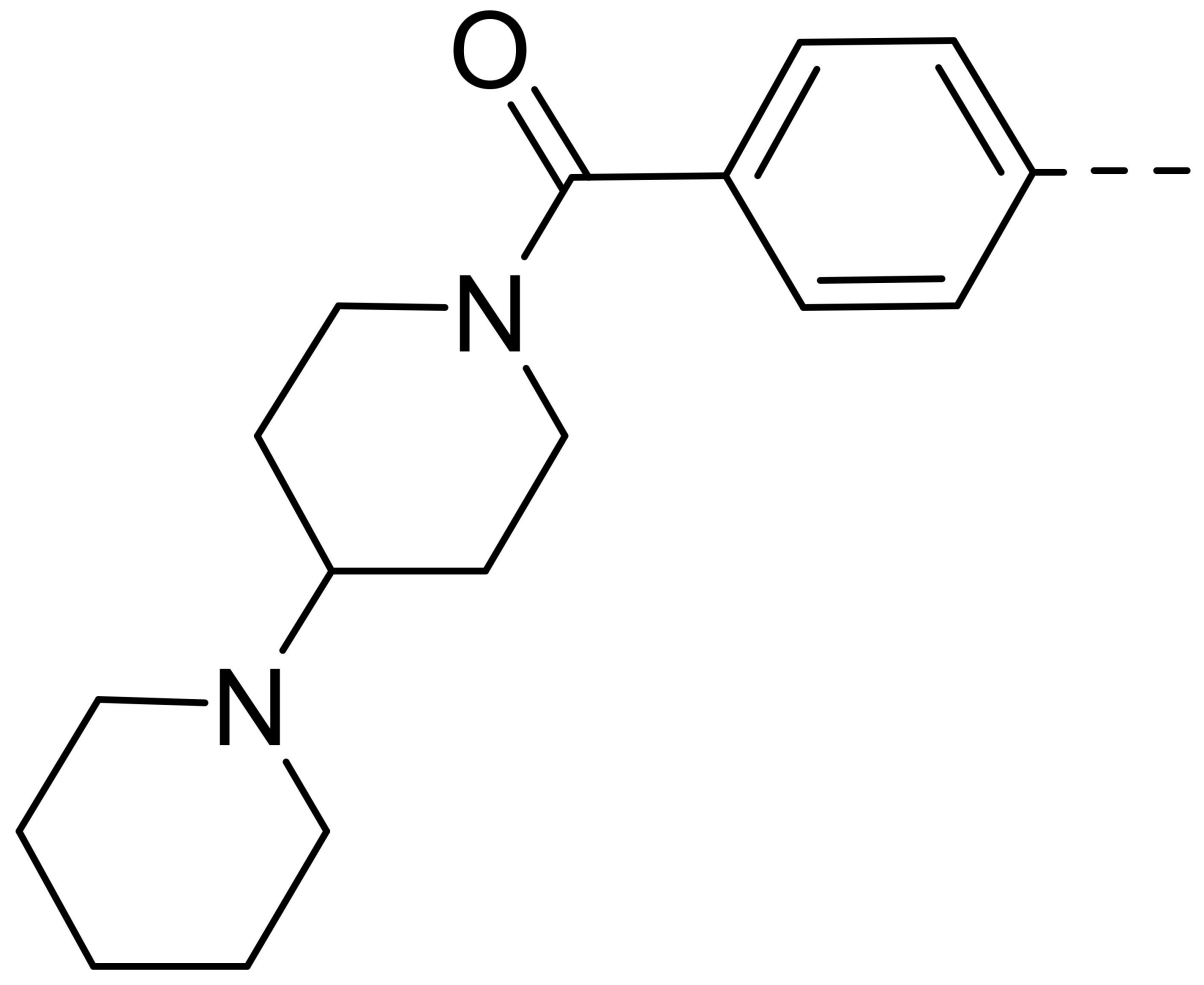	0%	**77**	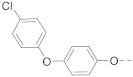	20%
**67**	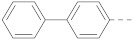	0%			

**Table 6:** Activities (IC_50_, μM) against the AF9/ENL AHD-AF4 interactions.

	AF9-DOT1L	AF9-AF4	ENL-AF4
**16**	2.7 ± 0.2	1.9 ± 0.1	1.9 ± 0.3
**24**	1.6 ± 0.2	2.1 ± 0.3	2.6 ± 0.3
**25**	5.3 ± 0.1	7.7 ± 0.9	7.9 ± 1.5
**50**	2.5 ± 0.2	2.5 ± 0.3	3.4 ± 0.4
**22**	>30	>50	>50
**37**	inactive	>50	>50

**Table 7:** Antitumor activity (EC_50_, μM) of selected compounds.

	MV4;11	Molm13	NB4	HL60	Hela
**2**	33 ± 2.8	29 ± 2.5	32 ± 2.0	50	>50
**12**	26 ± 1.8	50	50	50	>50
**15**	22 ± 2.0	10 ± 1.6	>50	50	>50
**24**	9.6 ± 1.2	4.7 ± 0.3	5.3 ± 1.1	10 ± 1.8	50
**25**	8.7 ± 0.9	9.4 ± 0.8	10 ± 0.5	26 ± 2.6	50
**50**	8.2 ± 1.2	5.1 ± 0.2	9.4 ± 1.2	9.0 ± 0.9	25 ± 2.5
**57**	21 ± 1.2	13 ± 1.7	11 ± 0.4	9.7 ± 1.2	50
**22**	>50	>50	>50	>50	>50
**37**	>50	>50	>50	>50	>50
**72**	~50	>50	>50	>50	>50

## Data Availability

Not applicable.

## Supplementary Materials

The following supporting information can be downloaded at: https://www.mdpi.com/article/10.3390/cancers15215283/s1, Figure S1. Dose-dependent inhibition of AF9 AHD-DOT1L interaction. Figure S2. Dose-dependent inhibition of different cancer cell growth.

## Abbreviations

MLL, mixed lineage leukemia; PPI, protein–protein interaction; MLL-r, mixed lineage leukemia-rearranged; onco-MLL, oncogenic fusion MLL; ALL, acute lymphocytic leukemia; AML, acute myeloid leukemia; SEC, super elongation complexes; H3K79, histone-H3 lysine-79; SAR, structure–activity relationship; BOC, *tert*-butyloxycarbonyl; SDS-PAGE, sodium dodecyl sulfate-polyacrylamide gel electrophoresis.
