# Supplementary material for: Discovery, Structure–Activity Relationship and In Vitro Anticancer Activity of Small-Molecule Inhibitors of the Protein–Protein Interactions between AF9/ENL and AF4 or DOT1L
Source: Cancers (Basel). 2023 Nov 3;15(21):5283. doi: 10.3390/cancers15215283 (PMC10650850; doi:10.3390/cancers15215283)

Supplementary Materials

Discovery, Structure–Activity Relationship and In Vitro Anticancer Activity of Small-Molecule Inhibitors of the Protein–Protein Interactions between AF9/ENL and AF4 or DOT1L

Xin Li, Xiaowei Wu, Shenyou Nie, Jidong Zhao, Yuan Yao, Fangrui Wu, Chandra Bhushan Mishra, Md Ashraf-Uz-Zaman, Bala Krishna Moku and Yongcheng Song

Table of Contents:

Figure S1 Dose-dependent inhibition of AF9 AHD-DOT1L interaction Page S2

Figure S2 Dose-dependent inhibition of different cancer cell growth Page S3

Respective HPLC tracers Page S4

The uncropped blots with densitometry readings/intensity ratio Page S7

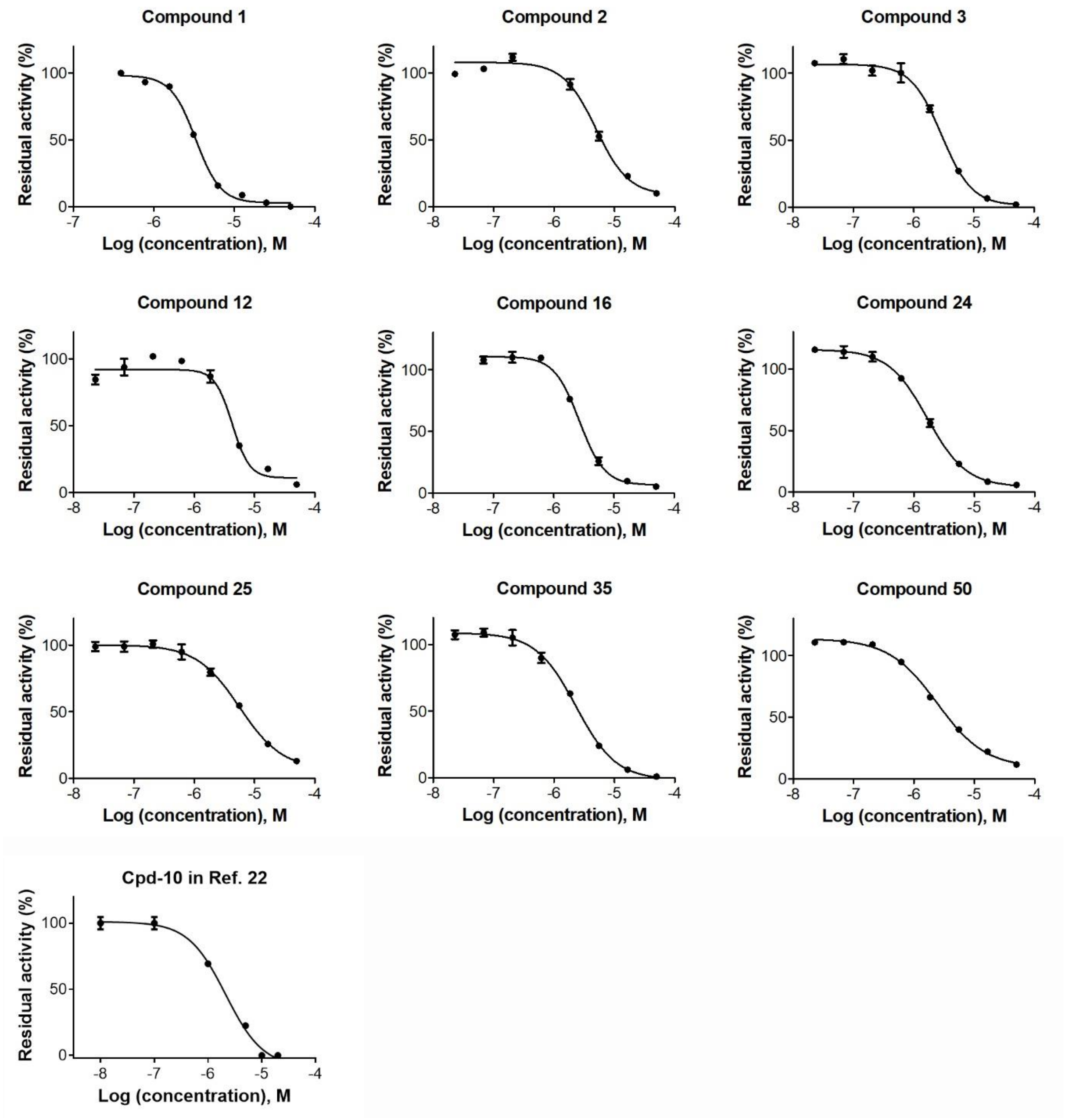

Figure S1 Dose-dependent inhibition of AF9 AHD-DOT1L interaction.

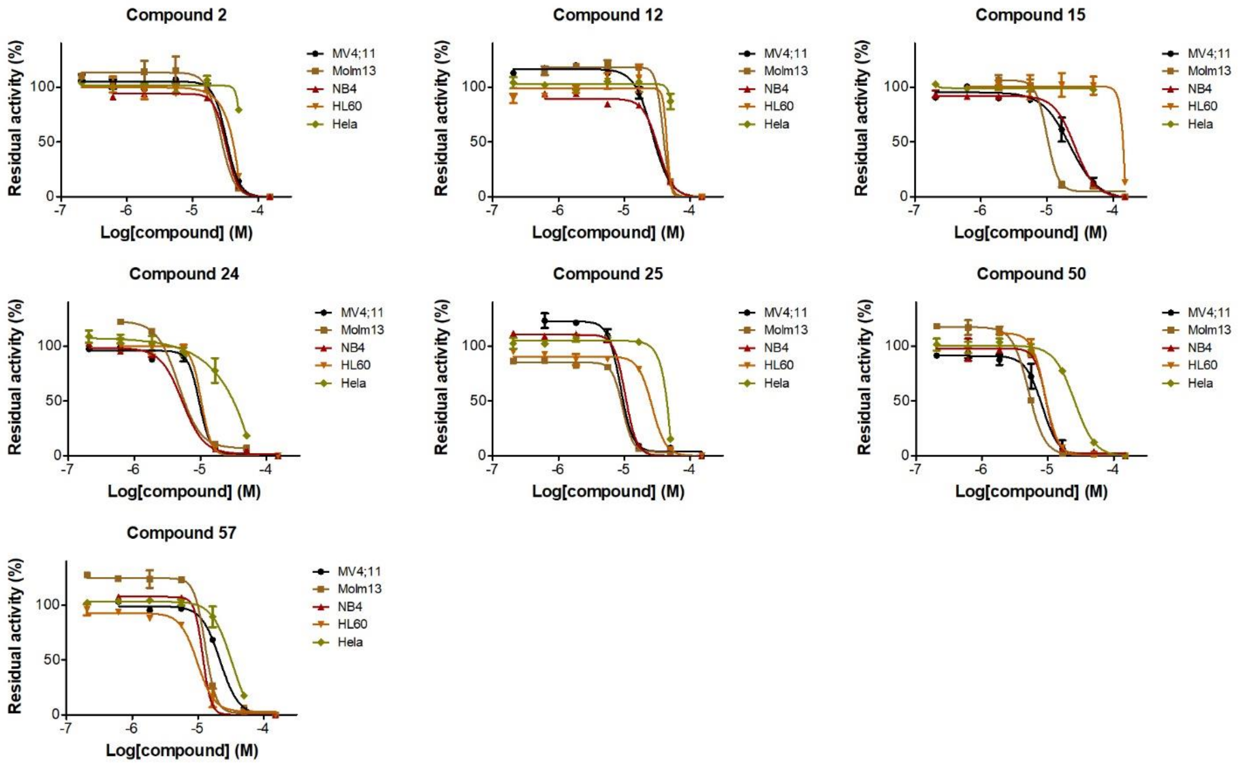

Figure S2 Dose-dependent inhibition of different cancer cell growth.

Respective HPLC tracers.

Compound 2, retention time, 3.232 min; MS (M + H)+ found 417.3

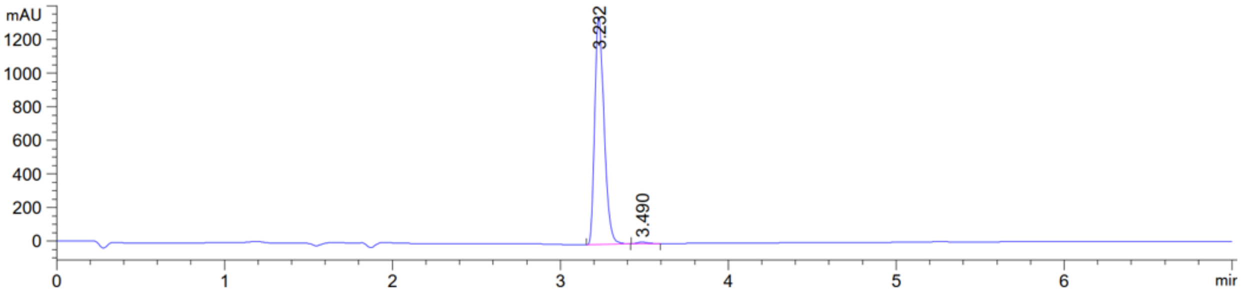

Signal 1: DAD1 A, Sig=254,4 Ref=off

Peak #	RetTime [min]	Type	Width [min]	Area [mAU*s]	Height [mAU]	Area %
1	3.232	BB	0.0588	5232.22998	1363.86230	99.1639
2	3.490	BB	0.0720	44.11741	9.89114	0.8361
Totals :				5276.34739	1373.75344	

Compound 12, retention time, 3.714 min; MS (M + H)+ found 403.3

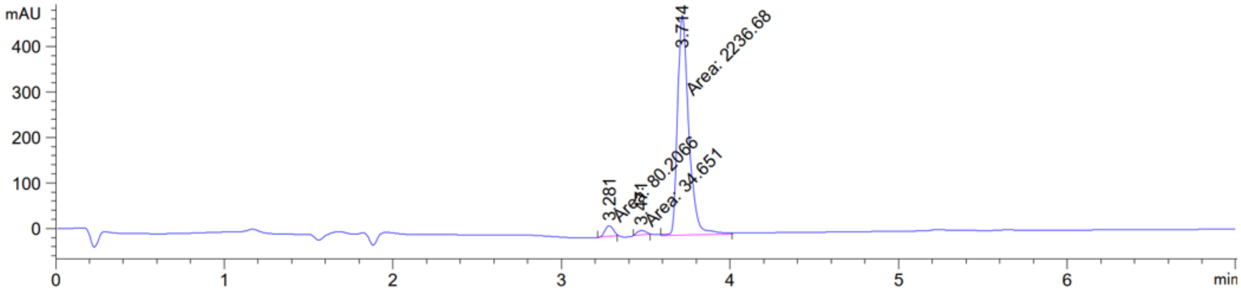

Signal 1: DAD1 A, Sig=254,4 Ref=off

Peak #	RetTime [min]	Type	Width [min]	Area [mAU*s]	Height [mAU]	Area %
1	3.281	MM	0.0566	80.20657	23.62328	3.4108
2	3.471	MM	0.0571	34.65096	10.11941	1.4735
3	3.714	MM	0.0766	2236.67993	486.57336	95.1156
Totals :				2351.53747	520.31605	

Compound 15, retention time, 3.335 min; MS (M + H)+ found 417.3

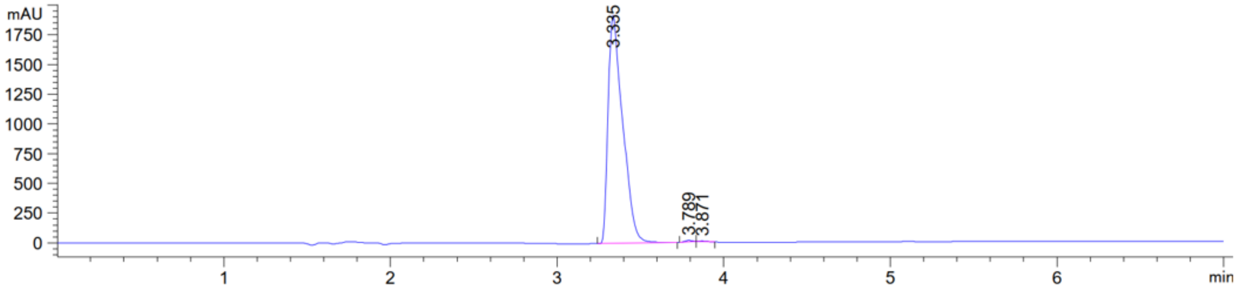

Signal 1: DAD1 A, Sig=254,4 Ref=off

Peak #	RetTime [min]	Type	Width [min]	Area [mAU*s]	Height [mAU]	Area %
1	3.335	BB	0.0867	1.16233e4	1917.57654	99.5664
2	3.789	BB	0.0497	36.38499	11.89926	0.3117
3	3.871	BB	0.0545	14.22750	4.31387	0.1219

Totals : 1.16739e4 1933.78967

Compound 16, retention time, 3.984 min; MS (M + H)+ found 403.3

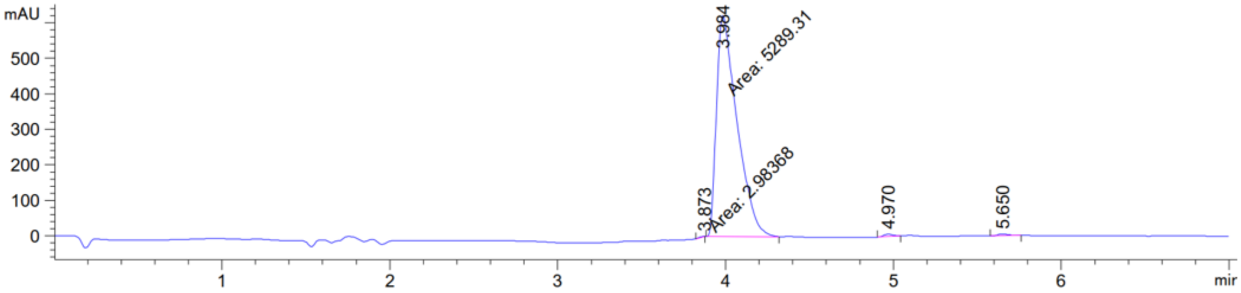

Signal 1: DAD1 A, Sig=254,4 Ref=off

Peak #	RetTime [min]	Type	Width [min]	Area [mAU*s]	Height [mAU]	Area %
1	3.873	MM	0.0496	2.98368	1.00349	0.0559
2	3.984	MM	0.1416	5289.30957	622.55463	99.0657
3	4.970	BB	0.0602	23.97388	6.33225	0.4490
4	5.650	BB	0.0675	22.92617	5.19708	0.4294

Totals : 5339.19330 635.08745

Compound 24, retention time, 3.484 min; MS (M + H)+ found 434.2

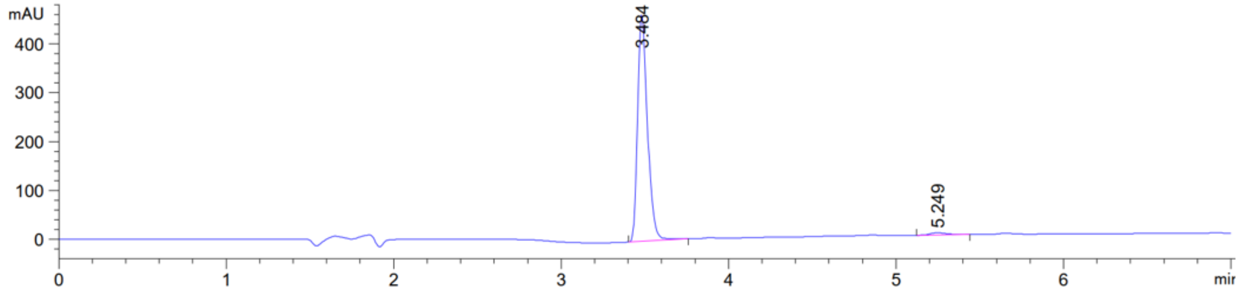

Signal 1: DAD1 A, Sig=254,4 Ref=off

Peak #	RetTime [min]	Type	Width [min]	Area [mAU*s]	Height [mAU]	Area %
1	3.484	BB	0.0623	1917.72510	463.20731	98.4394
2	5.249	BB	0.1029	30.40329	4.49297	1.5606

Totals : 1948.12839 467.70028

Compound 50, retention time, 3.458 min; MS (M + H)+ found 434.3

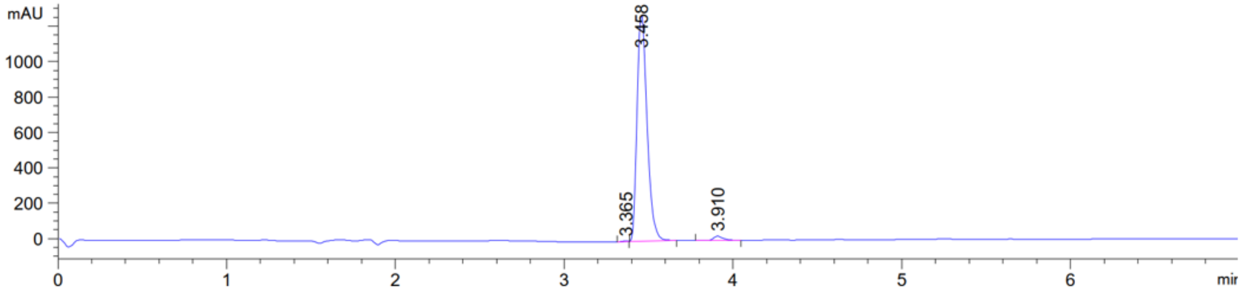

Signal 1: DAD1 A, Sig=254,4 Ref=off

Peak #	RetTime [min]	Type	Width [min]	Area [mAU*s]	Height [mAU]	Area %
1	3.365	BB	0.0403	5.05292	2.08479	0.0947
2	3.458	BB	0.0637	5231.61377	1279.34778	98.0551
3	3.910	BB	0.0623	98.71726	23.82796	1.8502

Totals : 5335.38396 1305.26054

The uncropped blots with densitometry readings/intensity ratio.

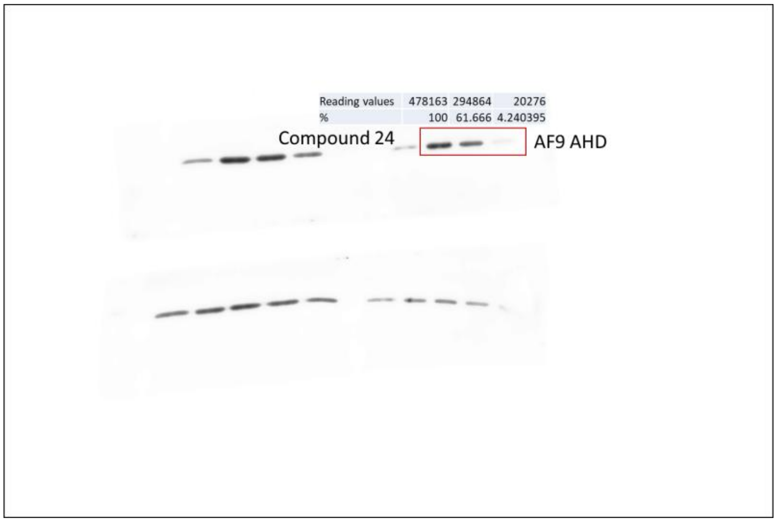

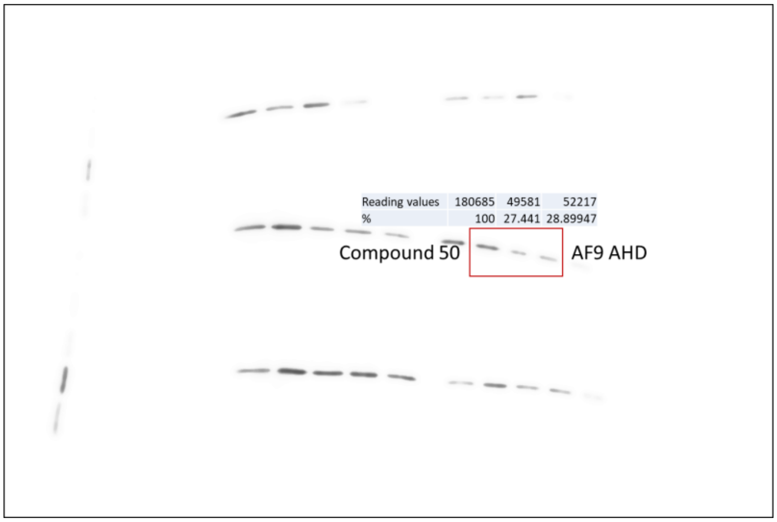

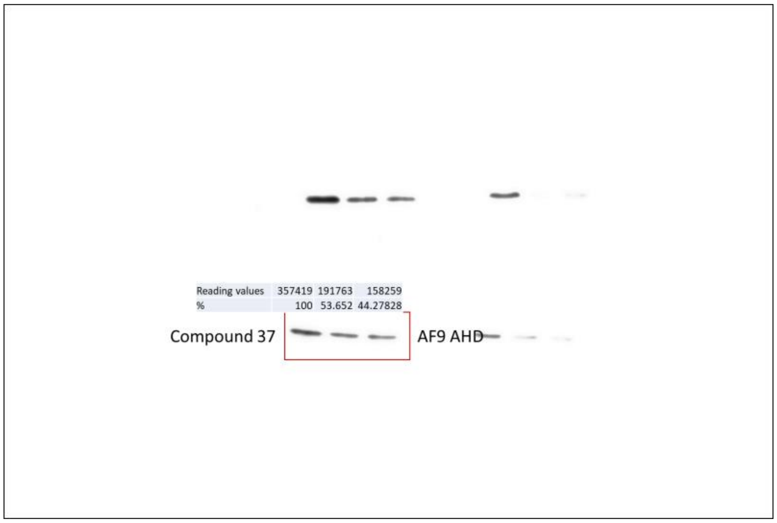

Supplement: Supplementary file 1 [file cancers-15-05283-s001.zip › cancers-2683376-supplementary.pdf]
